# Isolation of Nocuolin A and Synthesis of New Oxadiazine Derivatives. Design, Synthesis, Molecular Docking, Apoptotic Evaluation, and Cathepsin B Inhibition

**DOI:** 10.3390/md21050284

**Published:** 2023-04-29

**Authors:** Víctor Tena Pérez, Luis Apaza Ticona, Alfredo H. Cabanillas, Santiago Maderuelo Corral, Diego Fernando Rosero Valencia, Antera Martel Quintana, Montserrat Ortega Domenech, Ángel Rumbero Sánchez

**Affiliations:** 1 Department of Organic Chemistry, Faculty of Sciences, University Autónoma of Madrid, Cantoblanco, 28049 Madrid, Spain; 2 Organic Chemistry Unit, Department of Chemistry in Pharmaceutical Sciences, Faculty of Pharmacy, University Complutense of Madrid, Plza. Ramón y Cajal s/n, 28040 Madrid, Spain; 3 VALORALIA I MÁS D, SL, Tres Cantos, 28760 Madrid, Spain; 4 Spanish Bank of Algas, Institute of Oceanography and Global Change (IOCAG) University of Las Palmas de Gran Canarias, Muelle de Taliarte s/n, 35214 Telde, Canary Islands, Spain

**Keywords:** *Nostoc*, oxadiazines, anti-tumoural, cathepsins

## Abstract

Nocuolin A (**1**), an oxadiazine, was isolated from the cyanobacterium *Nostoc* sp. Its chemical structure was elucidated using NMR and mass spectroscopic data. From this compound, two new oxadiazines, 3-[(6*R*)-5,6-dihydro-4,6-dipentyl-2*H*-1,2,3-oxadiazin-2-yl]-3-oxopropyl acetate (**2**) and 4-{3-[(6*R*)-5,6-dihydro-4,6-dipentyl-2*H*-1,2,3-oxadiazin-2-yl]-3-oxopropoxy}-4-oxobutanoic acid (**3**), were synthesised. The chemical structures of these two compounds were elucidated by a combination of NMR and MS analysis. Compound **3** showed cytotoxicity against the ACHN (0.73 ± 0.10 μM) and Hepa-1c1c7 (0.91 ± 0.08 μM) tumour cell lines. Similarly, compound **3** significantly decreased cathepsin B activity in ACHN and Hepa-1c1c7 tumour cell lines at concentrations of 1.52 ± 0.13 nM and 1.76 ± 0.24 nM, respectively. In addition, compound **3** showed no in vivo toxicity in a murine model treated with a dose of 4 mg/kg body weight.

## 1. Introduction

Protein degradation, in both intracellular and extracellular spaces, is essential for the physiological equilibrium in healthy and diseased cells. It must, therefore, be strictly monitored [1]. Chronic diseases, especially cancer, are characterised by the dysregulation of proteolysis, which contributes to the progression of the disease [2]. Within this protein catabolism, lysosomal proteases have been identified. Among these proteases, several cysteine cathepsins are overexpressed in tumours [3]. 

Cathepsins are a large family of proteases composed of at least twelve different molecules that catalyse the hydrolysis of different proteins. There are three main types of enzymes depending on the amino acid of their active site, which can include a cysteine, aspartate, or a serine. Each of them has a unique structure, catalytic mechanism, and substrate specificity [4]. One of the degradation processes includes the macroautophagy pathway, where dysfunctional organelles are enclosed by a phagophore or isolation membrane (IM) that is later expanded for the creation of an autophagosome, a double-membrane vacuole that engulfs cellular components. Subsequently, the autophagosome fuses with lysosomes, where the lysosomal cathepsins, i.e., cathepsin B, D, L, etc., play a key role in allowing the normal function of the autophagosome [5]. This fusion gives rise to the creation of autolysosomes, which are essential structures for organelle degradation [6]. Additionally, cathepsin proteolytic activity has been directly linked with the determination of tumour cells' metastatic potential [7]. Furthermore, cysteinyl or aspartyl proteases can limit cell movement through the degradation of cellular matrices, which is related to migration and cell invasion [8]. Therefore, cathepsins are considered attractive targets for the development of new anticancer drugs. Cathepsins comprise a family of eleven proteases, out of which five (cathepsins B, H, K, L, and S) have been constantly involved in the progression of solid cancers [9,10]. 

According to the existing literature, many cancer types involve high levels of the protease cathepsin B. This abnormal concentration can be a result of a higher protein expression achieved by a higher number of copies of this gene, transcription rates, or more stable and/or more efficient mRNA variants [11]. Since high cathepsin B levels are directly related to cancer progression, cathepsin B has been proposed as a marker in cancer patients [12].

Thus, it is not surprising that the use of cathepsin B inhibitors can significantly reduce both metastases and tumour cell invasiveness, as has been already shown in several in vitro studies [13]. However, this process has also been related to the accumulation of lysosomal abnormal cathepsins (pro-cathepsins) that lead to the enlargement of lysosomes by an impaired autophagic process, the accumulation of these organelles, and the final cell death [14]. Therefore, these inhibitors can be a potential tool in cancer chemotherapy [15]. 

In this context, several cathepsin B synthetic inhibitors have been identified, such as organotellarides (IV), acyloxymethylketones, 1,2,4-thiadiazoles, aziridines, and epoxysuccinils; as well as those that come from natural sources, such as peptidyl aldehydes, aziridinyl peptides and epoxysuccinyl peptides, alkaloids, lactones, terpenes, and polyketides [16,17]. However, due to the resistance of some tumour cells to existing market drugs, more research is needed to identify new molecules with antitumour activity [18].

Within the natural sources, marine cyanobacteria occupy an important position due to their diverse structural types of more than 1600 metabolites, which can be grouped into 260 families [19]. This structural diversity has allowed the identification of a spectrum of pharmacological activities. Among these, nanomolar cytotoxicity (antitumour activity) against various tumour cell lines is one of the most relevant activities [20]. *Nostoc* sp. is a highly resistant new species that can be found in niches and can survive in diverse and extreme environmental conditions [21]. Extracts from *Nostoc* biomass have been used in the medical field for cancer therapy, immune stimulation, and antiviral activity. An example of an isolated compound from *Nostoc* with medical activity is a growth and proliferation suppressant polysaccharide (composed of xylose, arabinose, galactose, glucose, and mannose) in MCF-7 and DLD1 tumour cells [22].

In this article, we present how we isolated and characterised one compound from the *Nostoc* sp. species. Subsequently, we synthesised compounds starting from the most active compounds. Finally, we measured the antitumour activity, viability (Sulforhodamine B), and cathepsin B inhibition of natural and synthetic compounds in ACHN (*Mus musculus* kidney carcinoma, CRL-1611), Hepa-1c1c7 (*Mus musculus* liver carcinoma, CRL-2026) as tumour cell lines, and TKPTS (*Mus musculus* kidney normal, CRL-3361) and FL83B (*Mus musculus* liver normal, CRL-2390) as non-tumour cell lines. 

## 2. Results and Discussion

### 2.1. Structural Elucidation of the Natural Compound

The planar structure of **1** was deduced by the analysis of HRESIMS, 1D, and 2D NMR experiments as 1-[(6*R*)-5,6-dihydro-4,6-dipentyl-2*H*-1,2,3-oxadiazin-2-yl]-3-hydroxypropan-1-one (Supplementary Materials). 

**Figure 1:**
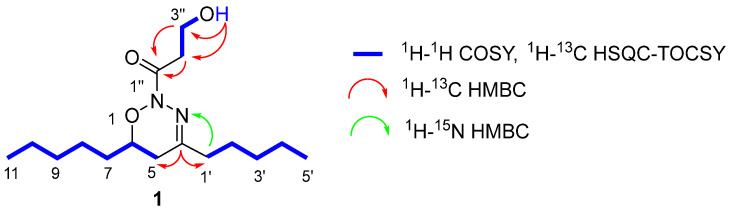
^1^H-^1^H COSY, ^1^H-^13^C HSQC-TOCSY connectivity and most representatives ^1^H-^13^C HMBC connectivity for compound **1**.

Compound **1** (Figure 1) was patented by the company VALORALIA I MÁS D, SL [23]. However, in the same month of filing the acceptance of the patent process, a patent was made public in the Czech Republic, describing exactly the same compound, which they named Nocuolin A. Subsequently, the same research group published an article with the structural characterisation and antitumour activity of compound **1** [24]. On the other hand, the compound Nocuolin A has also been reported in the species *Nodularia* sp. [25]. Despite this, the company VALORALIA I MÁS D, SL has ownership of the exploitation of the uses of the invention in European, Asian, and American countries.

### 2.2. Synthesis of Analogues of the Natural Compound

**Scheme 1:**
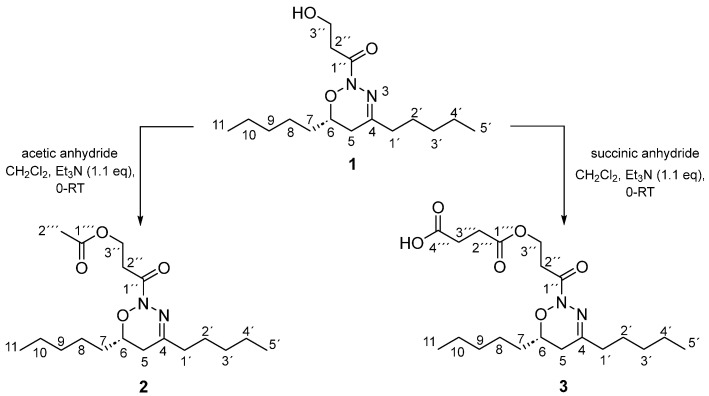
Chemical synthesis of analogues **2** and **3** from compound **1**.

The analogues doxadiazine 2 (compound **2**) and doxadiazine 3 (compound **3**) were synthesised from compound **1** by esterification reactions. In this sense, the reaction of compound **1** with acetic anhydride in trimethylamine yielded 88% of compound **2** (colourless oil). On the other hand, to obtain compound **3**, compound **1** was esterified with succinic anhydride and trimethylamine, obtaining it with a 60% yield and a colourless oil (Scheme 1).

The elucidation of the structures of derivatives **2** and **3** was carried out by comparison with the 1D and 2D spectra in CDCl_3_ of compound **1**. The complete 1D NMR (^1^H and ^13^C) assignment of compound **1** was possible only after recording several 1D and 2D (^1^H-^1^H COSY, ^1^H-^13^C HSQC, ^1^H-^13^C HSQC-TOCSY, ^1^H-^13^C HMBC and ^1^H-^15^N HMBC) in CDCl_3_ at 500 MHz (Figures S9–S16). Subsequently, the NMR spectral data of both derivatives **2** and **3** were compared with compound **1**’s data (see experimental section).

Compound **2,** which was obtained by synthesis from compound **1** as a colourless oil, gave the molecular formula of C_18_H_32_N_2_O_4_, obtained by HRESIMS at *m*/*z* 363.2444 (calcd. for C_18_H_32_NaN_2_O_4_, 363.2435) (Figure S17). The analysis of the 1D NMR spectra (^1^H, ^13^C, and DEPT-135) (Figures S18–S20) suggested that the only difference between the 1D NMR spectra of compound **2** and the 1D NMR spectra of compound **1** was the presence of one acetate group.

More concretely, methyl protons at *δ*_H_ 2.02 (s) and *δ*_C_ 21.1, and quaternary carbon at *δ*_C_ 171.1. In addition, the chemical shift of methylene protons H-3’’ at *δ* 4.40 (t, *J* = 6.5 Hz) showed the structure of compound **2** as 3-[(6*R*)-5,6-dihydro-4,6-dipentyl-2*H*-1,2,3-oxadiazin-2-yl]-3-oxopropyl acetate (Scheme 1).

Compound **3** had the molecular formula of C_20_H_34_N_2_O_6_, which was determined in the same way as for compound **1**, via HRESIMS based on the [M + Na]^+^ ion peak at *m*/*z* 421.2304 (calcd. for C_20_H_34_NaN_2_O_6_, *m*/*z* 421.2309) (Figure S21). Analysis of 1D NMR (^1^H, ^13^C, and DEPT) and 2D NMR (^1^H-^1^H COSY, ^1^H-^13^C HSQC, and ^1^H-^13^C HMBC) (Figures S22–S27) suggested that compound **3** is similar structurally to compound **1**, except two new methylene groups at *δ*_H_ 2.62 (t, *J* = 6.5 Hz, 2H) and *δ*_H_ 2.63 (t, *J* = 6.5 Hz, 2H), and two quaternary carbons at *δ*_C_ 177.1 and 172.1. ^1^H-^13^C HMBC showed a connection between the protons H-2’’’ and H-3’’’ with the carbon atoms C-1’’’/C-4’’’, and the protons H-3’’ at *δ* 4.43 (t, *J* = 6.5 Hz) with the quaternary carbon C-1’’ (*δ* 164.8) and C-1’’’ (*δ* 172.1). Thus, this suggested the structure of compound **3** as 4-{3-[(6*R*)-5,6-Dihydro-4,6-dipentyl-2*H*-1,2,3-oxadiazin-2-yl]-3-oxopropoxy}-4-oxobutanoic acid (Scheme 1).

Compounds **2** and **3**, named Doxadiazine 2 and Doxadiazine 3, respectively, were patented by the VALORALIA I MÁS D, SL company, having ownership of the uses of the invention in countries of Europe, Asia, and America [26].

### 2.3. In Vitro Viability

**Figure 2:**
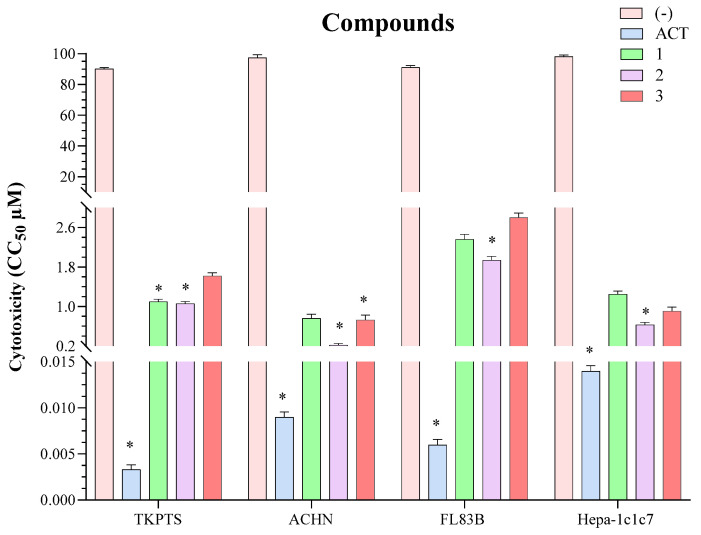
Calculated SRB (viability) assay for compounds **1**–**3** in two non-tumour cell lines (TKPTS and FL83B) and two tumour cell lines (ACHN and Hepa-1c1c7) at 72 h. ACT (Actinomycin D). Confidence interval 95% (CI_95_%), * p < 0.001 (Tukey’s multiple comparisons test).

Figure 2 shows the cytotoxicity of the pure compounds compared with Actinomycin as a positive control that showed CC_50_ values of 0.003 ± 0.0005 and 0.006 ± 0.0006 μM for the non-tumour cell lines TKPTS and FL83B. Likewise, the positive control showed CC_50_ values of 0.009 ± 0.0006 and 0.014 ± 0.0006 μM for the tumour cell lines ACHN and Hepa-1c1c7.

Compounds **1** and **3** did not show significant cytotoxicity compared to Actinomycin in the TKPTS non-tumour cell lines (CC_50_ of 1.10 ± 0.05 and 1.62 ± 0.06 μM, respectively) and FL83B (CC_50_ of 2.36 ± 0.10 and 2.80 ± 0.09 μM, respectively). Concerning their cytotoxicity on tumour cell lines, compounds **1** and **3** showed CC_50_ values of 0.76 ± 0.09 and 0.73 ± 0.10 μM (ACHN cells) and CC_50_ of 1.25 ± 0.06 and 0.91 ± 0.08 μM (Hepa-1c1c7 cells).

On the other hand, compound **2** presented higher cytotoxicity than compounds **1** and **3** on tumour cell lines ACHN and Hepa-1c1c7 with CC_50_ values of 0.22 ± 0.03 and 0.63 ± 0.05 μM, respectively. However, compound **2** also showed significant cytotoxicity against the non-tumour cell lines TKPTS and FL83B with CC_50_ values of 1.06 ± 0.04 and 1.94 ± 0.08 μM, respectively (Figure 2).

### 2.4. Inhibition of Cathepsin B Activity 

**Figure 3:**
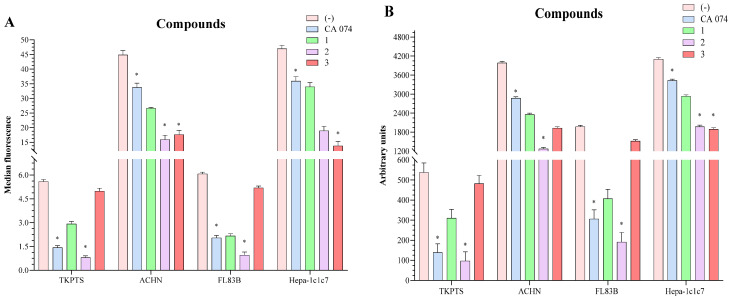
Cathepsin B activity in non-tumour cells and tumour cells. (**A**) Quantitative cytofluorimetric analysis of plasma membrane cathepsin B in two non-tumour cell lines (TKPTS and FL83B) and two tumour cell lines (ACHN and Hepa-1c1c7). (**B**) Fluorometric assays for cathepsin B activity in the cell medium of four representative cell lines of tumour and non-tumour cells. Results are reported as fluorescence units. Confidence interval 95% (CI_95_%), * p < 0.001 (Tukey’s multiple comparisons test).

For the determination of the cathepsin B’s inhibition activity, its superficial expression in two tumour cell lines (ACHN and Hepa-1c1c7 cells) and two non-tumour cell lines (TKPTS and FL83B cells) was evaluated by flow cytometry. CA 074 is an inhibitor of cathepsin B and was used as a positive control with *K_i_* values of 2.39 ± 0.12 and 3.08 ± 0.19 nM for the non-tumour cell lines TKPTS and FL83B. Likewise, the positive control showed *K_i_* values of 5.62 ± 0.73 and 5.99 ± 0.64 nM for the tumour cell lines ACHN and Hepa-1c1c7 (Figure 3A).

Compound **1** decreased the amount of cathepsin B on tumour cell lines similarly to the positive control (*p* = 0.5374), but with lower *K_i_*, 3.06 ± 0.13 nM (ACHN cells) and 3.37 ± 0.24 nM (Hepa-1c1c7 cells). However, compound **1** also decreased the amount of cathepsin B in the FL83B non-tumour cell line (*p* = 0.8511), with similar results to the positive control, obtaining a *K_i_* of 2.61 ± 0.02 nM. However, this last effect was smaller on the non-tumour cell line TKPTS at a *K_i_* of 2.13 ± 0.04 nM (*p* < 0.001, compared to the positive control) (Figure 3A).

Compounds **2** and **3** showed a higher decrease in the amount of cathepsin B from tumour cell lines than the positive control (*p* < 0.001). However, compound **3** showed this effect at a lower *K_i_* (*K_i_* = 1.52 ± 0.13 nM and *K_i_* = 1.76 ± 0.24 nM, in ACHN cells and Hepa-1c1c7 cells, respectively) than the positive control. On the other hand, compound **2** showed a similar effect (*K_i_* = 5.67 ± 0.68 nM, ACHN cells and *K_i_* = 6.59 ± 0.65 nM, Hepa-1c1c7 cells) to the positive control (Figure 3A).

Although compound **2** showed the potential of decreasing the amount of cathepsin B on tumour cell lines, it was observed that it also significantly affected non-tumour cell lines compared to the positive control (*p* < 0.001), reporting *K_i_* of 0.49 ± 0.05 nM (TKPTS cells) and 0.66 ± 0.09 nM (FL83B cells). In the case of compound **3**, it did not substantially decrease the amount of cathepsin B in non-tumoral cell lines (*p* < 0.001), presenting higher *K_i_* (*K_i_* = 3.29 ± 0.40 nM, TKPTS cells and *K_i_* = 4.55 ± 0.81nM, FL83B cells) (Figure 3A).

Figure 3B shows the results obtained in both tumour cell lines (ACHN and Hepa-1c1c7 cells) and two non-tumour cell lines (TKPTS and FL83B cells) in relation to cathepsin B’s activity, which was higher against tumour cells than non-tumour ones (*p* < 0.001).

Cathepsin B is a 30-kDa, bilobal protein with an active site and substrate-binding cleft located in the interface of a left (L-) and a right (R-) domain with three and two binding sites located in the loops, categorised as S3, S1, and S2’, and S1’ and S2, respectively. Access of the substrate into the active site is controlled by the occluding loop, which consists of an 18-residue-long insertion (Pro 107-Asp 124) [27].

Based on the structure of this enzyme, it is known that the catalytic triad of cathepsin B is formed by a cysteine, a histidine, and an aspartic acid. The Cys29 and His199 residues interact, catalysing the cleavage of the peptide bond. Therefore, cathepsin B inhibitors must have an electrophilic character capable of reacting with the thiol group of the Cys29 residue [17]. Examples include synthetic ‘warheads’ such as aldehydes, disulphides, vinyl sulfones, and halomethyl ketones [28]. In our work, we used the compound CA 074 as a positive control, whose mechanism of action is performed through its binding to the S’ subsites in a similar direction as the substrate [17].

All the compounds (one isolated and two synthesised) share the essential premise of maintaining a main oxadiazine scaffold, which is structurally similar to thiadiazols. In this sense, a cleavage of the N-O bond of oxadiazine by the thiolate group of cathepsin B could produce the opening of the ring and proteolytic enzymatic inhibition [17].

Although there are reports that compound **1** (1-[(6*R*)-5,6-dihydro-4,6-dipentyl-2*H*-1,2,3-oxadiazin-2-yl]-3-hydroxypropan-1-one) promotes apoptosis in lung, uterine, pancreatic, ovarian, breast, and brain tumour cell lines, inhibiting caspase at concentrations between 0.72 and 4.50 μM [24], its effect in other cancers through the cathepsin B inhibition has not been studied. It is in this sense that our study contributes by showing that the inhibition activity of compound **1** is superior to the positive control (CA 074).

However, in the case of compound **2**, to improve the membrane permeability, an ester-type derivative was introduced. Nevertheless, the activity results obtained were worse (loss of selectivity) compared to compound **1** and the positive control. These data agree with the consulted bibliography since the esterification of CA 074 (Prodrug) improves its permeability through cell membranes but decreases its activity and selectivity [29].

Finally, biological assays showed that compound **3** was the most active of the three compounds. Analysing its structure, we observed that the presence of a free carboxylate group improved the selectivity for cysteine proteases in comparison to other nucleophilic biological sulphides [17]. In addition, the presence of this free carboxylate group allowed interactions with the His111 residue of the occlusion loop, improving the selectivity of the compounds [30].

Although previous in vitro studies suggest that compound **3** may be a potential cathepsin B inhibitor, additional molecular coupling analyses are required for a better understanding of the molecule–enzyme interaction. 

### 2.5. Molecular Docking of the Active Compounds

To confirm the interactions of compounds (**1**–**3**) and of the positive control (CA 074) with the cathepsin B protease (PDB: 1QDQ), in silico docking studies were performed using GOLD software [31], which deploys a genetic algorithm (GA) to generate different ligand conformations and defines the binding site using the reference inhibitor CA 074, using a cut-off distance of 0.8 Å.

**Figure 4:**
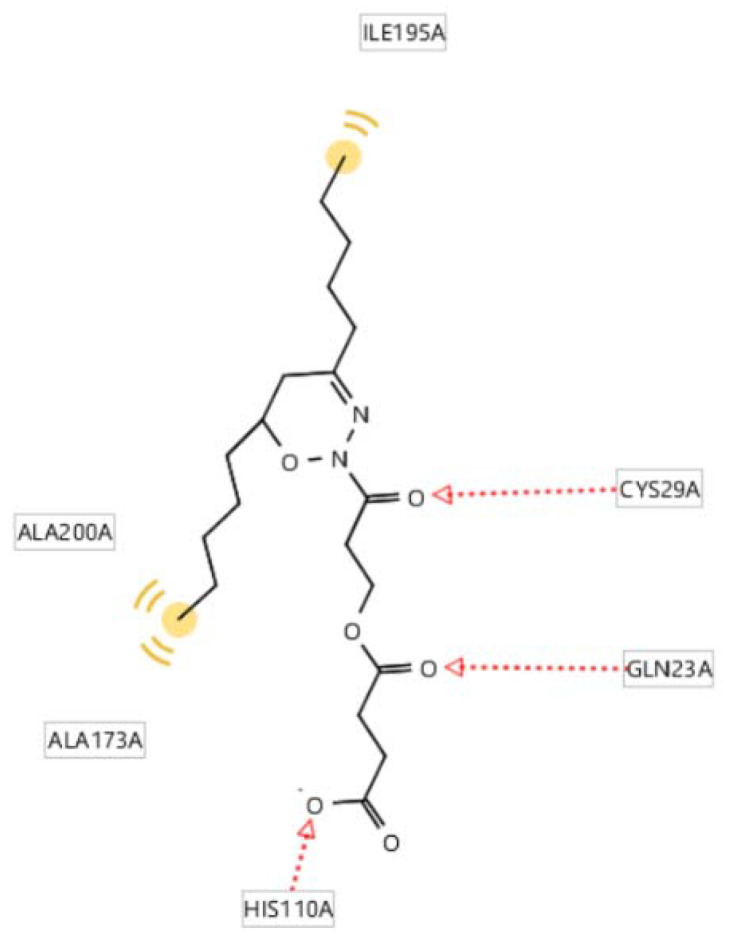
A 2D diagram displaying hydrophobic interactions (represented in yellow) and hydrogen bond acceptors (indicated by red arrows) of compound **3**.

On the other hand, docking solutions were evaluated with the ChemPLP scoring function [32] and the GoldScore re-scoring function [33]. Subsequently, the best-scored poses of the most populated groups were selected for further analysis, and the ones that did not include interactions with essential residues for the enzymatic activity were discarded. It is important to highlight that while crystallography provides detailed information about ligand binding, docking provides guidance on what that binding might look like and should not be taken as definitive since there may be variations, for example, in hydrophobic interactions depending on the position of the alkyl tails. Therefore, a more detailed interpretation must be supported by further binding studies (Figure 4).

**Figure 5:**
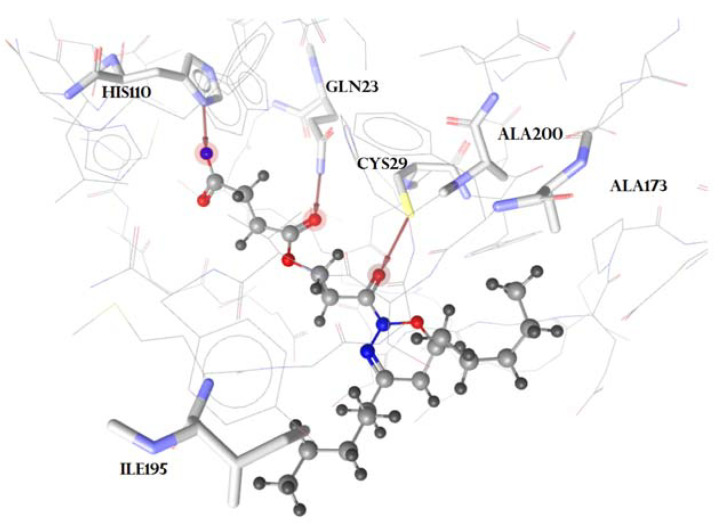
Compound **3** at the binding site, forming hydrogen bonds with residues (highlighted in red) previously identified as crucial for cathepsin inhibitions interactions.

Analysis of the interactions between compound **3** and cathepsin B revealed the formation of hydrogen bonds between Cys29A, Gln29A, and His110 and the molecule, in addition to hydrophobic interactions with other residues such as Ala200A. This cysteine-29 residue is part of the catalytic triad responsible for enzyme activity [34] and, typically, inhibitors must have an electrophilic character capable of reacting with cysteine residue 29 [17]. This suggests that compound **3** binds similarly to the reference inhibitor CA 074 and other compounds described previously [27,35]. In addition, the carboxylate group present in compound **3** establishes interactions with the His110A residue, which has also been described as an important residue for the binding of ligands to cathepsin B [36] (Figure 5).

**Figure 6:**
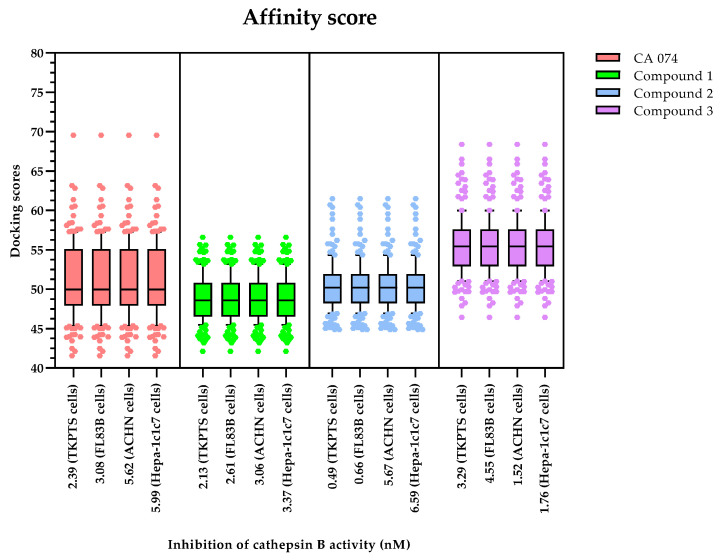
Affinity of compounds (**1**–**3**) and positive control (CA 074) on cathepsin B.

Finally, a correlation was made between the coupling scores (obtained from 300 different poses and evaluated through the affinity (PLP score) and rescore (GOLD) functions) and the inhibition constants (*Ki*) of the samples (Figure 6).

Using the box and whisker plot, we can conclude that there is not a perfect correlation between the coupling scores and the experimental *Ki*. This tells us that the exact binding mode of each compound to cathepsin B needs to be further investigated through structural biology experiments.

### 2.6. Acute Toxicity In Vivo

Based on the preliminary positive results of in vitro cytotoxicity, we decided to carry out in vivo toxicological studies at the same dose as the positive control, CA 074 (4 mg/kg), to determine whether the compounds showed unwanted effects on mice. For this, mice were divided into five groups, which were given different treatments based on the presence of the positive control and compounds **1**, **2,** or **3** (groups two, three, four, and five, respectively). After the third day of treatment, mice in groups two (4 mg/kg *i.p*., CA 074), three (4 mg/kg *i.p.,* compound **1**), and five (4 mg/kg *i.p.,* compound **3**) were active and showed no apparent weight loss. However, in group four (4 mg/kg *i.p.,* compound **2**), weight loss and signs of disease (bowed backs, loss of hair shine, and slow movements) were observed.

It was not possible to administer higher doses because this study aimed to determine whether the compounds were toxic at the same concentration as the positive control (CA 074), so the LD_50_ values were not determined and the degree of involvement of the mice was measured subjectively on a gradual scale from 0 to 10; where higher values represent an increase in physical harm observed in animals, including death.

**Figure 7:**
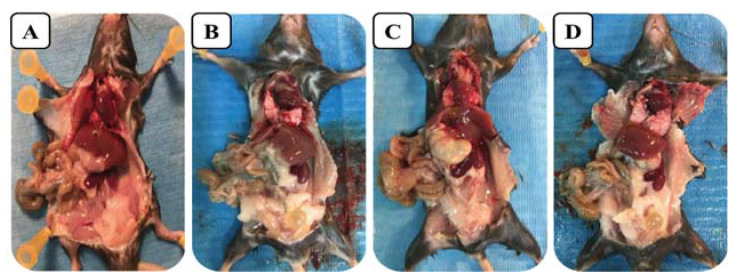
Necroscopy of the different groups of mice under study after one week of treatment. (**A**) group two treated with CA 074 (4 mg/kg *i.p*.); (**B**) group three treated with the natural compound (**1**) (4 mg/kg *i*.*p*.); (**C**) group four treated with compound **2** (4 mg/kg *i*.*p*.); (**D**) group five treated with compound **3** (4 mg/kg *i*.*p*.).

After concluding the week of treatment, major signs of toxicity were not detected (macroscopic observation). The animals were sacrificed, and histological brain, intestine, heart, liver, kidney, and pancreas sections were performed. Histological analysis of mice in all groups showed no inflammation, necrosis, fibrosis, or hyperplasia signs. Even group four, where mice showed signs of disease on the third day, revealed no signs of toxicity or damaged organs (Figure 7).

## 3. Material and Methods

### 3.1. General Experimental Procedures

First-grade organic solvents, purchased from Sigma-Aldrich, were used for the isolation of the compounds. Thin-layer chromatography (TLC) was performed using Merck Silica gel 60-F_254_ plates. The different chromatoplates were visualised by UV absorbance (254 nm) and stained with phosphomolybdic acid followed by heating. A silica gel (40–63 μm and 20–45 μm, Merck) chromatography column was prepared using the indicated eluent in accordance with standard techniques. 

NMR experiments were performed with Bruker Avance DRX 300 and 500 spectrometers operating at 300 MHz/500 MHz (^1^H) or 75 MHz/175 MHz (^13^C). Chloroform-*d*_1_ and DMSO-*d*_6_ were used as deuterated solvents. The spectra were calibrated by assigning the peaks of the residual solvents. HREIMS spectra were obtained through electronic impact techniques (EI+), at 70 e.V., and electrospray (ESI+), using a QSTAR XL quadrupole TOF mass spectrometer. MS samples were prepared in MeOH. 

### 3.2. Biological Material 

A sample of the cyanobacterium *Nostoc* sp. (BEA0798B) was collected from Garajonay National Park, La Gomera, Gran Canarias (Canary Islands, 28°07′40.7″N and 17°14′12.6″W, Spain). The 16S ribosomal RNA gene sequence was deposited in GenBank (accession No. OQ379060.1). The sample was cultured in 2 L flasks with BG-110 medium under axenic conditions by VALORALIA I MÁS D, SL. Cultures were grown with an air bubbling at 23 ± 1 °C and ambient light for 21 days. After this time, the biomass generated was separated by centrifugation and, finally, lyophilised.

### 3.3. Extraction and Isolation

The lyophilised biomass (15.1 g) was extracted by repeated maceration with CH_2_Cl_2_/MeOH (1:1, 3 × 1 L) to obtain a dichloromethane/methanol extract (3.17 g). The biological activity (apoptotic capacity and cathepsin B inhibition) of this extract was measured against tumour and non-tumour cell lines.

The dichloromethane/methanol extract (3 g) was fractionated in a column chromatography (12 × 60 cm) with silica gel (40–63 μm), using as the mobile phases a hept/AcOEt (1:1, 3 × 500 mL), CH_2_Cl_2_/MeOH (20:1, 3 × 500 mL), and CH_2_Cl_2_/MeOH (5:1, 3 × 500 mL) to produce four fractions (A–D). Fraction **C** (337 mg) was found to be the most active fraction (apoptotic capacity and cathepsin B inhibition).

Finally, fraction **C** (300 mg) was subjected to separation by silica gel (20–45 μm) column (8 × 30 cm) chromatography, using hept/AcOEt (1:1, 250 mL) as an eluent, obtaining the sub-fractions I (100 mg), II (5 mg), **III** (54 mg), IV (17 mg), and V (161 mg). Subsequently, sub-fraction **C-III** (50 mg) was purified by silica gel (20–45 μm) column (8 × 30 cm) chromatography, using hept/AcOEt (1:2, 100 mL) as an eluent, obtaining compound **1** (31 mg).

### 3.4. General Procedure for the Synthesis of Oxadiazines Analogues

Initially, a solution of the natural compound (10 mg, 0.03 mmol, 1.0 eq.) in CH_2_Cl_2_ at 0 °C, Et_3_N (4 µL, 0.036 mmol, 1.1 eq.) was added, followed by acetic anhydride (3 µL, 0.036 mmol, 1.1 eq.). Performing the steps described above, compound **2** (colourless oil) obtained an 88% yield after purification by column chromatography with hept/AcOEt (2:1).

In parallel, a solution of the natural compound (13 mg, 0.044 mmol, 1.0 eq.) in CH_2_Cl_2_ at 0 °C, Et_3_N (7 µL, 0.048 mmol, 1.1 eq.) was added, followed by succinic anhydride (5 mg, 0.048 mmol, 1.1 equiv.). The mixture was kept stirring for 5 h. After testing the end of the reaction by TLC, the solvent was distilled under reduced pressure. The remaining oily residue was purified by column chromatography with hept/AcOEt (1:2), obtaining a colourless oily compound (**3**).

### 3.5. Spectroscopic Data

#### 3.5.1. 1-[(6*R*)-5,6-Dihydro-4,6-dipentyl-2*H*-1,2,3-oxadiazin-2-yl]-3-hydroxypropan-1-one (**1**)

Colourless viscous oil; [α${]}_{D}^{25}$ = −75.0° (*c* = 0.033, MeOH); IR *ν*_máx_ 3410, 2953.3, 2926.3, 2856.0, 1726.9, 1671.6, 1627.7, 1461.8, 1401.1, 1380.9, 1048.4, 998.6, 943.9, 725.3 cm^−1^; ^1^H NMR (500 MHz, CDCl_3_): *δ*_H_ 4.05 (dddd, *J* = 8.8, 7.8, 4.9, 3.8 Hz, 1H, H-6), 3.91 (t, *J* = 6.6 Hz, 2H, H-3’’), 2.98 (br, 1H, H3’’-OH), 2.86 (t, *J* = 6.6 Hz, 2H, H-2’’), 2.30 (dd, *J* = 18.1, 3.8 Hz, 1H, H-5a), 2.30 (t, *J* = 6.6 Hz, 2H, H-1’), 2.21 (dd, *J* = 18.1, 8.8 Hz, 1H, H-5b), 1.71 (m, 1H, H-7a), 1.56 (m, 2H, H-2’), 1.51 (m, 1H, H-7b), 1.40 (m, 2H, H-8), 1.35-1.30 (m, 8H, H-9, H-10, H-3’, H-4’), 0.90 (t, *J* = 6.8 Hz, 3H, H-5’), 0.89 (t, *J* = 6.8 Hz, 3H, H-11); ^1^H NMR (500 MHz, DMSO): *δ*_H_ 4.00 (dddd, *J* = 8.8, 7.8, 4.8, 3.8 Hz, 1H, H-6), 3.66 (td, *J* = 6.6, 5.4 Hz, 2H, H-3’’), 4.57 (t, *J* = 5.4 Hz, 1H, H3’’-OH), 2.69 (m, *J* = 6.6 Hz, 1H, H-2’’a), 2.68 (m, *J* = 6.6 Hz, 1H, H-2’’b), 2.39 (dd, *J* = 18.3, 3.8 Hz, 1H, H-5a), 2.23 (t, *J* = 7.3 Hz, 1H, H-1’a), 2.22 (t, *J* = 7.3 Hz, 1H, H-1’b), 2.17 (dd, *J* = 18.3, 8.8 Hz, 1H, H-5b), 1.54 (m, 1H, H-7a), 1.53 (m, 2H, H-2’), 1.48 (m, 1H, H-7b), 1.36 (m, 2H, H-8), 1.30–1.27 (m, 8H, H-9, H-10, H-3’, H-4’), 0.88 (t, *J* = 7.3 Hz, 3H, H-5’), 0.87 (t, *J* = 6.8 Hz, 3H, H-11); ^13^C NMR (175 MHz, CDCl_3_): *δ*_C_ 167.1 (C-1’’), 150.7 (C-4, determined by HMBC), 75.6 (C-6), 58.6 (C-3’’), 37.5 (C-1’), 36.1 (C-2’’), 34.1 (C-7), 31.8 (C-5), 31.7 (C-9), 31.5 (C-3´), 25.7 (C-2’), 24.5 (C-8), 22.6 (C-10), 22.5 (C-4’), 14.1 (C-11), 14.1 (C-5’); ^13^C NMR (175 MHz, DMSO): *δ*_C_ 165.6 (C-1’’), 150.8 (C-4, determined by HMBC), 75.3 (C-6), 57.4 (C-3’’), 36.3 (C-1’), 37.3 (C-2’’), 33.7 (C-7), 31.5 (C-5), 31.5 (C-9), 31.2 (C-3´), 25.3 (C-2’), 24.3 (C-8), 22.6 (C-10), 22.4 (C-4’), 14.3 (C-11), 14.3 (C-5’); HRESIMS *m*/*z* [M + Na]^+^ 301.2152 (calcd. for C_16_H_30_NaN_2_O_3_ 301.2149). 

#### 3.5.2. 3-[(6*R*)-5,6-Dihydro-4,6-dipentyl-2*H*-1,2,3-oxadiazin-2-yl]-3-oxopropyl Acetate (**2**)

Colourless oily; [α${]}_{D}^{25}$ = −50.2° (*c* = 0.064, MeOH); ^1^H NMR (300 MHz, CDCl_3_): *δ*_H_ 4.40 (t, *J* = 6.5 Hz, 2H, H-3’’), 4.03 (dddd, *J* = 8.8, 7.8, 4.9, 3.8 Hz, 1H, H-6), 2.93 (t, *J-* = 6.5 Hz, 2H, H-2’’), 2.28 (dd, *J* = 18.1, 3.8 Hz, 1H, H5a), 2.23 (t, *J* = 6.6 Hz, 2H, H1’), 2.14 (dd, 1H, *J* = 18.1, 8.8 Hz, H-5b), 1.70 (m,1H, H-7a), 1.52 (m, 2H, H-2’), 1.50 (m, 1H, H-7b), 1.43 (m, 2H, H-8), 1.30-1.20 (m, 8H, H-9, H-10, H-3’, H-4´), 0.90 (t, *J* = 6.5 Hz, 3H, H-5’), 0.87 (t, *J* = 6.5 Hz, 3H, H-11); ^13^C NMR (75 MHz, CDCl_3_): *δ*_C_ 171.1 (C-1’’’), 164.9 (C-1’’), 150.4 (C-4, determined by HMBC), 75.5 (C-6), 60.2 (C-3’’), 37.2 (C-1’), 3.1 (C-7), 33.3 (C-2’’), 31.8 (C-5/C-3’), 31.4 (C-9), 25.8 (C-2’), 24.4 (C-8), 22.6 (C-10), 22.5 (C-4’), 21.1 (C-2’’’), 14.1 (C-11/C-5’); HRESIMS *m*/*z* [M]^+^ 363.2444 (calcd. for C_18_H_33_NaN_2_O_4_ 363.2435). 

#### 3.5.3. 4-{3-[(6*R*)-5,6-Dihydro-4,6-dipentyl-2*H*-1,2,3-oxadiazin-2-yl]-3-oxopropoxy}-4-oxobutanoic Acid (**3**)

Colourless oily; [α${]}_{D}^{25}$ = −63.2° (*c* = 0.047, MeOH); ^1^H NMR (300 MHz, CDCl_3_): *δ*_H_ 4.43 (t, *J* = 6.5 Hz, 2H, H-3’’), 4.04 (dddd, *J* = 8.8, 7.8, 4.9, 3.8 Hz, 1H, H-6), 2.95 (t, *J* = 6.4 Hz, 2H, H-2’’), 2.63 (t, *J* = 6.5 Hz, 4H, H-2’’’/H-3’’’), 2.28 (dd, *J* = 18.1, 3.8 Hz, 1H, H-5a), 2.24 (t, *J* = 6.6 Hz, 2H, H-1’), 2.15 (dd, *J* = 18.1, 8.8 Hz, 1H, H-5b), 1.68 (m, 1H, H-7a), 1.50 (m, 2H, H-2’), 1.48 (m, 1H, H-7b), 1.25 (m, 8H, H-9, H-10, H-3’, H-4’), 0.91 (t, *J* = 6.6 Hz, 3H, H-5’), 0.88 (t, *J* = 6.6 Hz, 3H, H11); ^13^C NMR (75 MHz, CDCl_3_): *δ*_C_ 177.1 (C-4’’’), 172.1 (C-1’’’), 164.8 (C-1’’), 150.4 (C-4, determined by HMBC), 75.5 (C-6), 60.5 (C-3’’), 37.2 (C-1’), 33.9 (C-7), 33.2 (C-2’’), 31.6 (C-5/C-3’), 31.4 (C-9), 28.8 (C-2’’’/C-3’’’), 25.5 (C-2’), 24.6 (C-8), 22.3 (C-10/C-4’), 14.0 (C-11/C-5’); HRESIMS *m*/*z* [M + Na]^+^ 421.2304 (calcd. for C_20_H_34_NaN_2_O_6_ 421.2309). 

### 3.6. Cell Culture

Two tumour cell lines, ACHN (*Human kidney* carcinoma) and Hepa-1c1c7 (*Human* liver carcinoma), and two non-tumour cell lines, TKPTS (*Mus musculus* kidney normal) and FL83B (*Mus musculus* liver normal), were used in this study. The cell lines were purchased by the company VALORALIA I MÁS D, SL. From LGC Standards, which is the exclusive distributor of American Type Culture Collection (ATTC) products. The data provided by the company indicate that ACHN (CRL-1611 BSL 1) is a cell line exhibiting epithelial morphology that was isolated in 1979 from the kidney of a male patient with adenocarcinoma. Likewise, Hepa-1c1c7 (CRL-2026 BSL 1) is an epithelial cell line that was isolated from the liver of a patient with hepatoma. On the other hand, TKPTS (CRL-3361 BSL 2) is a normal cell line with epithelial-like morphology that was isolated in 1997 from the proximal tubule of a mouse kidney. Finally, FL83B (CRL-2390 BSL 1) is a hepatocyte cell that was isolated from the liver of a 15- to 17-day-old foetal mouse in 1969. The cells were maintained in a Dulbecco's modified eagle medium (DMEM, Sigma-Aldrich St. Louis, USA) supplemented with 2 mM L-glutamine (AppliChem, Denmark), 10% foetal bovine serum (FBS, Sigma-Aldrich, USA), 100 units/mL of penicillin and 100 µg/mL of streptomycin (Fisher Scientific, Pittsburgh, USA) in culture flasks, in an incubator with a humidified atmosphere containing 5% CO_2_, at 37 °C under hypoxic conditions (1% O_2_), managing to mimic the tumour microenvironment in vivo.

### 3.7. Viability Assay

Cells (200 µL, 2 × 10^4^ cells/well) were seeded in 96-well plates and were cultured for 12 h at 37 °C under hypoxic conditions (1% O_2_). Subsequently, the samples dissolved in dimethyl sulfoxide (DMSO ≥ 99.9% Sigma-Aldrich, CAS Number 67-68-5) were added at different concentrations for 72 h. Actinomycin D (ACT ≥ 95% Sigma-Aldrich, CAS Number 50-76-0) was used as a positive control [37]. Cells were then fixed with 10% trichloroacetic acid (TCA ≥ 99.0% Sigma-Aldrich, CAS Number 76-03-9) for 1 h at 4 °C. After washing with distilled water, 100 μL of 0.4% sulforhodamine B (SRB Dye content 75 % Sigma-Aldrich, CAS Number 3520-42-1) in 1% acetic acid (AcOH ≥ 99.0% Sigma-Aldrich, CAS Number 64-19-7) were added to each well and incubated for 20 min. After washing them three times with 1% TCA, the plates were air-dried, and stained cells were dissolved with 100 μL/well of 10 mM unbuffered tris(hydroxymethyl)aminomethane (Tris Base ≥ 99.9% Sigma-Aldrich, CAS Number 77-86-1). The optical density of the microplates was measured at 540 nm, using an ELISA plate reader, SpectraMax^®^ i3, Molecular Devices. 

### 3.8. Assay for Activity of Cathepsin B

Cathepsin B activity in the cell medium was evaluated by using a fluorogenic substrate: Abz-Gly-Ile-Val-Arg~Ala-Lys(Dnp)-OH. This peptide substrate was purchased from Bachem AG (Bubendorf, Switzerland). The reactions were performed at 40 °C for 10 min [38]. Stock solutions of inhibitory compounds were prepared in DMSO (in a concentration of 10 mM). From these stock solutions, increasing concentrations of the inhibitory compounds (1 and 100 nM) were prepared. L-3-*trans*-(propylcarbamyl)-oxirane-2-carbonyl)-L-isoleucyl-L-proline (CA 074 ≥ 99%Sigma-Aldrich, CAS number 134448-10-5) was used as a positive control [39]. Fluorescence was measured with excitation/emission wavelength values of 320 and 420 nm, respectively, using the Cary Eclipse fluorescence spectrophotometer (Agilent Technologies, Melbourne, Australia). 

### 3.9. In Vivo Toxicity

Adult male C57BL/6 mice weighing 25–35 g were purchased from the Centre for Laboratory Animals of the Faculty of Medicine from the University Autónoma of Madrid (Spain) and maintained under controlled temperature conditions (22 ± 2 °C), with a constant 12 h light-dark cycle and with access to food and water ad libitum. The experiments reported in this study were performed in accordance with the guidelines of the EU Directive 2010/63/EU for experiments on animals and Royal Decree 53/2013/Spain. These experiments were approved by the Institutional Ethics Committee of the Faculty of Medicine of the University Autónoma of Madrid (Protocol No. 125601 of 4 June 2018).

Five groups (five mice per group) were selected for this study. Group one served as a negative control and received a normal saline solution at a dose of 5 mL/kg (*i*.*p*.), group two served as a positive control and received compound CA 074 at a concentration of 4 mg/kg, *i.p*. [40], group three were treated with the natural compound (**1**) (4 mg/kg, *i*.*p*.), group four were treated with compound **2** (4 mg/kg, *i*.*p*.), and group five were treated with compound **3** (4 mg/kg, *i*.*p*.). The concentration of 4 mg/kg of the natural compound and the two synthesised compounds were chosen based on the concentration reported for compound CA 074. This is because our study aimed to compare the toxicity of our compounds with compound CA 074, which is a cathepsin B inhibitor that reduces tumour cell metastasis.

All animals were kept under observation for 7 days, during which their physical aspect, behaviour, and the number of deaths were recorded daily. The negative control (normal saline solution) received only the same quantity of the vehicle (distilled water). These tests were carried out in triplicate. All the surviving animals were euthanised at the end of the study, and their vital organs were individually observed by necropsy and checked for overt pathology.

### 3.10. Statistical Analysis

All analyses were performed using version 9.0.0 of GraphPad Prism Software LLC from 1994–2020 (www.graphpad.com, accessed on 2 January 2023). Using non-linear regression, the values of 50% cytotoxic concentration (CC_50_) and 50% inhibitory concentration (IC_50_) were determined. In addition, a one-way ANOVA statistical analysis was performed to assess whether the differences between the values were statistically significant (*p* < 0.05; *p* < 0.001, Tukey’s multiple comparisons test). All experiments were performed in triplicate.

### 3.11. Computational Methods

In silico docking studies for compounds **1**–**3** and the positive control (CA 074) were carried out using GOLD software [31] (Hermes 2022.2.0 (Build 353591)). The protocol was validated by removing the co-crystallised ligand of the receptor, and by re-docking it into the active site (RMSD was used for evaluation of results). For energy-minimised ligand docking, 300 GA runs, and 125,000 operations were used. 

## 4. Conclusions

In conclusion, this study described the isolation of the natural oxadiazine 1-[(6*R*)-5,6-Dihydro-4,6-dipentyl-2*H*-1,2,3-oxadiazin-2-yl]-3-hydroxypropan-1-one from the strain *Nostoc* sp. (BEA0798B), and through a series of modifications of its chemical structure, two new oxadiazines were synthesised. It was shown that the derivative 4-{3-[(6*R*)-5,6-Dihydro-4,6-dipentyl-2*H*-1,2,3-oxadiazin-2-yl]-3-oxopropoxy}-4-oxobutanoic acid (compound **3**) showed cytotoxicity on tumour lines ACHN and Hepa-1c1c7 at concentrations of 0.73 ± 0.10 μM and 0.91 ± 0.08 μM, respectively. Likewise, this derivative showed inhibition of cathepsin B activity in tumour cells at concentrations of 1.52 ± 0.13 nM (ACHN cells) and 1.76 ± 0.24 nM (Hepa-1c1c7 cells). Furthermore, this derivative did not show toxicity in vivo (murine model) when a dose of 4 mg/kg *i.p.* was used. All these findings suggest that this derivative exhibits important anticancer potential against in vitro tumour cells and in vivo assays for hepatocellular (HCC) and renal cell carcinoma (RCC) models.

## Data Availability

The original data presented in the study are included in the article/Supplementary Material; further inquiries can be directed to the corresponding author.

## Supplementary Materials

The following supporting information can be downloaded at: https://www.mdpi.com/article/10.3390/md21050284/s1, NMR and HRESIMS spectra of the synthetic oxadiazines analysed in this study are provided as supporting information (Figures S1–S27).
