# Supplementary material for: Isolation of Nocuolin A and Synthesis of New Oxadiazine Derivatives. Design, Synthesis, Molecular Docking, Apoptotic Evaluation, and Cathepsin B Inhibition
Source: Mar Drugs. 2023 Apr 29;21(5):284. doi: 10.3390/md21050284 (PMC10221466; doi:10.3390/md21050284)
Supplement: Supplementary file 1 [file marinedrugs-21-00284-s001.zip › marinedrugs-2339257-supplementary.pdf]

Supplementary Materials

Isolation of Nocuolin A and synthesis of new oxadiazine derivatives. Design, synthesis, molecular docking, apoptotic evaluation and cathepsin B inhibition

Víctor Tena Pérez[1], Luis Apaza Ticona[1,2,*], Alfredo H. Cabanillas[1], Santiago Maderuelo Corral[3], Diego Fernando Rosero Valencia[3], Antera Martel Quintana[4], Montserrat Ortega Domenech[3], Ángel Rumbero Sánchez[1,*]

Affiliation

1. Department of Organic Chemistry, Faculty of Sciences, University Autónoma of Madrid, Cantoblanco, 28049 Madrid, Spain
2. Organic Chemistry Unit, Department of Chemistry in Pharmaceutical Sciences, Faculty of Pharmacy, University Complutense of Madrid, Plza. Ramón y Cajal s/n, 28040 Madrid, Spain
3. VALORALIA I MÁS D, SL, Tres Cantos, 28760 Madrid, Spain
4. Spanish Bank of Algas, Institute of Oceanography and Global Change (IOCAG) University of Las Palmas de Gran Canarias, Muelle de Taliarte s/n, 35214 Telde, Canary Islands, Spain

***Correspondence:**

lnapaza@ucm.es (L.A.T). Organic Chemistry Unit, Department of Chemistry in Pharmaceutical Sciences, Faculty of Pharmacy, University Complutense of Madrid. Plza. Ramón y Cajal s/n, 28040 Madrid, Spain. ORCID: 0000-0002-7135-3909

angel.rumbero@uam.es (A.R.S). Department of Organic Chemistry, Faculty of Sciences, University Autónoma of Madrid. Cantoblanco, 28049 Madrid, Spain

Contents:

- Structural elucidation of 1-[(6*R*)-5,6-Dihydro-4,6-dipentyl-2*H*-1,2,3-oxadiazin-2-yl]-3-hydroxypropan-1-one (**1**).
- **Table S1.** ^{1}H-^{1}H COSY, ^{1}H-^{13}C HSQC-TOCSY connectivity and most representatives ^{1}H-^{13}C HMBC connectivity for 1-[(6*R*)-5,6-Dihydro-4,6-dipentyl-2*H*-1,2,3-oxadiazin-2-yl]-3-hydroxypropan-1-one (**1**).
- **Figure S1**. HRESIMS spectrum of 1-[(6*R*)-5,6-Dihydro-4,6-dipentyl-2*H*-1,2,3-oxadiazin-2-yl]-3-hydroxypropan-1-one (**1**).
- **Figure S2**. ^{1}H NMR spectrum of 1-[(6*R*)-5,6-Dihydro-4,6-dipentyl-2*H*-1,2,3-oxadiazin-2-yl]-3-hydroxypropan-1-one (**1**) in DMSO 500 MHz.
- **Figure S3**. ^{13}C NMR spectrum of 1-[(6*R*)-5,6-Dihydro-4,6-dipentyl-2*H*-1,2,3-oxadiazin-2-yl]-3-hydroxypropan-1-one (**1**) in DMSO 175 MHz.
- **Figure S4**. DEPT-135 spectrum of 1-[(6*R*)-5,6-Dihydro-4,6-dipentyl-2*H*-1,2,3-oxadiazin-2-yl]-3-hydroxypropan-1-one (**1**) in DMSO 175 MHz.
- **Figure S5**. ^{1}H-^{1}H COSY spectrum of 1-[(6*R*)-5,6-Dihydro-4,6-dipentyl-2*H*-1,2,3-oxadiazin-2-yl]-3-hydroxypropan-1-one (**1**) in DMSO.
- **Figure S6**. ^{1}H-^{13}C HSQC spectrum of 1-[(6*R*)-5,6-Dihydro-4,6-dipentyl-2*H*-1,2,3-oxadiazin-2-yl]-3-hydroxypropan-1-one (**1**) in DMSO.
- **Figure S7**. ^{1}H-^{13}C HMBC spectrum of 1-[(6*R*)-5,6-Dihydro-4,6-dipentyl-2*H*-1,2,3-oxadiazin-2-yl]-3-hydroxypropan-1-one (**1**) in DMSO.
- **Figure S8**. ^{1}H-^{15}N HMBC spectrum of 1-[(6*R*)-5,6-Dihydro-4,6-dipentyl-2*H*-1,2,3-oxadiazin-2-yl]-3-hydroxypropan-1-one (**1**) in DMSO.
- **Figure S9**. ^{1}H NMR spectrum of 1-[(6*R*)-5,6-Dihydro-4,6-dipentyl-2*H*-1,2,3-oxadiazin-2-yl]-3-hydroxypropan-1-one (**1**) in $CDCl_3$ 500 MHz.
- **Figure S10**. ^{13}C NMR spectrum of 1-[(6*R*)-5,6-Dihydro-4,6-dipentyl-2*H*-1,2,3-oxadiazin-2-yl]-3-hydroxypropan-1-one (**1**) in $CDCl_3$ 175 MHz.
- **Figure S11**. DEPT-135 spectrum of 1-[(6*R*)-5,6-Dihydro-4,6-dipentyl-2*H*-1,2,3-oxadiazin-2-yl]-3-hydroxypropan-1-one (**1**) in $CDCl_3$ 175 MHz.
- **Figure S12**. ^{1}H-^{1}H COSY spectrum of 1-[(6*R*)-5,6-Dihydro-4,6-dipentyl-2*H*-1,2,3-oxadiazin-2-yl]-3-hydroxypropan-1-one (**1**) in $CDCl_3$.
- **Figure S13**. ^{1}H-^{13}C HSQC spectrum of 1-[(6*R*)-5,6-Dihydro-4,6-dipentyl-2*H*-1,2,3-oxadiazin-2-yl]-3-hydroxypropan-1-one (**1**) in $CDCl_3$.
- **Figure S14**. ^{1}H-^{13}C HMBC spectrum of 1-[(6*R*)-5,6-Dihydro-4,6-dipentyl-2*H*-1,2,3-oxadiazin-2-yl]-3-hydroxypropan-1-one (**1**) in $CDCl_3$.
- **Figure S15**. ^{1}H-^{13}C HSQC-TOCSY spectrum of 1-[(6*R*)-5,6-Dihydro-4,6-dipentyl-2*H*-1,2,3-oxadiazin-2-yl]-3-hydroxypropan-1-one (**1**) in $CDCl_3$.
- **Figure S16**. ^{1}H-^{15}N HMBC spectrum of 1-[(6*R*)-5,6-Dihydro-4,6-dipentyl-2*H*-1,2,3-oxadiazin-2-yl]-3-hydroxypropan-1-one (**1**) in $CDCl_3$.
- **Figure S17**. HRESIMS spectrum of 3-[(6*R*)-5,6-Dihydro-4,6-dipentyl-2*H*-1,2,3-oxadiazin-2-yl]-3-oxopropyl acetate (**2**).
- **Figure S18**. ^{1}H NMR spectrum of 3-[(6*R*)-5,6-Dihydro-4,6-dipentyl-2*H*-1,2,3-oxadiazin-2-yl]-3-oxopropyl acetate (**2**) in $CDCl_3$ 300 MHz.
- **Figure S19**. ^{13}C NMR spectrum of 3-[(6*R*)-5,6-Dihydro-4,6-dipentyl-2*H*-1,2,3-oxadiazin-2-yl]-3-oxopropyl acetate (**2**) in $CDCl_3$ 75 MHz.
- **Figure S20**. DEPT-135 spectrum of 3-[(6*R*)-5,6-Dihydro-4,6-dipentyl-2*H*-1,2,3-oxadiazin-2-yl]-3-oxopropyl acetate (**2**) in $CDCl_3$ 75 MHz.
- **Figure S21**. HRESIMS spectrum of 4-{3-[(6*R*)-5,6-Dihydro-4,6-dipentyl-2*H*-1,2,3-oxadiazin-2-yl]-3-oxopropoxy}-4-oxobutanoic acid (**3**).
- **Figure S22**. ^{1}H NMR spectrum of 4-{3-[(6*R*)-5,6-Dihydro-4,6-dipentyl-2*H*-1,2,3-oxadiazin-2-yl]-3-oxopropoxy}-4-oxobutanoic acid (**3**) in $CDCl_3$ 300 MHz.

- **Figure S23**. ^{13}C NMR spectrum of 4-{3-[(6*R*)-5,6-Dihydro-4,6-dipentyl-2*H*-1,2,3-oxadiazin-2-yl]-3-oxopropoxy}-4-oxobutanoic acid (**3**) in $CDCl_3$ 75 MHz.
- **Figure S24**. DEPT-135 spectrum of 4-{3-[(6*R*)-5,6-Dihydro-4,6-dipentyl-2*H*-1,2,3-oxadiazin-2-yl]-3-oxopropoxy}-4-oxobutanoic acid (**3**) in $CDCl_3$ 75 MHz.
- **Figure S25**. ^{1}H-^{1}H COSY spectrum of 4-{3-[(6*R*)-5,6-Dihydro-4,6-dipentyl-2*H*-1,2,3-oxadiazin-2-yl]-3-oxopropoxy}-4-oxobutanoic acid (**3**) in $CDCl_3$.
- **Figure S26**. ^{1}H-^{13}C HSQC spectrum of 4-{3-[(6*R*)-5,6-Dihydro-4,6-dipentyl-2*H*-1,2,3-oxadiazin-2-yl]-3-oxopropoxy}-4-oxobutanoic acid (**3**) in $CDCl_3$.
- **Figure S27**. ^{1}H-^{13}C HMBC spectrum of 4-{3-[(6*R*)-5,6-Dihydro-4,6-dipentyl-2*H*-1,2,3-oxadiazin-2-yl]-3-oxopropoxy}-4-oxobutanoic acid (**3**) in $CDCl_3$.

- Structural elucidation of 1-[(6*R*)-5,6-Dihydro-4,6-dipentyl-2*H*-1,2,3-oxadiazin-2-yl]-3-hydroxypropan-1-one (**1**).

Compound **1** was isolated as a colourless viscous oil, with $C_{16}H_{30}N_2O_3$ as a molecular formula based on the $[M + Na]^+$ ion peak at *m/z* 321.2152 (calcd. for $C_{16}H_{30}NaN_2O_3$ 321.2149) obtained by HRESIMS (Figure S1). ^1H NMR (Figure S2) in DMSO-d_6 showed the presence of the hydroxyl proton at δ_H 4.57 (t, *J* = 5.4 Hz) which coupled by a ^1H-^1H COSY correlation with methylene protons at δ_H 3.66 (td, *J* = 6.6 and 5.4 Hz, H-3′′) and δ_C 57.4 determined by ^{13}C NMR (Figure S3) and DEPT-135 (Figure S4) spectra. The ^1H-^1H COSY (Figure S5) and ^1H-^{13}C HSQC (Figure S6) spectra showed that the methylene CH_2-3′′ was correlated with the methylene CH_2-2′′ at δ_H 2.69 (t, *J* = 6.6 Hz, 1H, Ha-2′′), 2.68 (t, *J* = 6.6 Hz, 1H, Hb-2′′) and δ_C 37.3. The methylene CH_2-2′′ and CH_2-3′′ of spin system A were interconnected to the quaternary carbon at δ_C 165.6 (C-1′′) as shown by the ^1H-^{13}C HMBC spectrum (Figure S7) which suggested an amide bond (N-CO-CH_2-CH_2OH). Other two spin systems in the ^1H NMR spectrum could be identified in the ^1H-^1H COSY, ^1H-^{13}C HSQC and ^1H-^{13}C HMBC spectra as follows: CH_3-CH_2-CH_2-CH_2-CH_2 (C-1′/C-5′, spin system B) and CH_3-CH_2-CH_2-CH_2-CH_2-CHR-CH_2 (C-5/C-11, spin system C). The spin system B and C were seen as interconnected via attachment to the quaternary carbon at δ_C 150.2 (C-4) by ^1H-^{13}C HMBC correlations between C-4 with methylene protons H-5 at δ_H 2.39 (dd, *J* = 18.3 and 3.8 Hz, H-5a), 2.17 (dd, *J* = 18.3 and 8.8 Hz, H-5b) and with methylene protons H-1′ at δ_H 2.23 (t, *J* = 7.3 Hz, H-1′a), 2.22 (t, *J* = 7.3 Hz, H-1′b). The correlation between H-1′ with a nitrogen atom at δ_N 302.6 which was observed in the ^1H-^{15}N HMBC (Figure S8) showed the presence of an imine bond between C-4 and N-3 atoms. Finally, the signals at δ_H 4.00 (dddd, *J* = 8.8, 7.8, 4.8 and 3.8 Hz) and δ_C 75.3 were assigned to methane oxygenated CH-6, using the ^1H-^1H COSY correlations with methylene protons CH_2-5 and CH_2-7 at δ_H 1.54 (m, H-7a), 1.48 (m, H-7b) and δ_C 33.7 (C-7). All assignments of ^1H and ^{13}C NMR in DMSO-d_6 of the compound are shown in Table S1.

- **Table S1.** ^1H-^1H COSY, ^1H-^{13}C HSQC-TOCSY connectivity and most representatives ^1H-^{13}C HMBC connectivity for 1-[(6*R*)-5,6-Dihydro-4,6-dipentyl-2*H*-1,2,3-oxadiazin-2-yl]-3-hydroxypropan-1-one (**1**) in DMSO-d_6.

No	δ_C, DEPT-135	δ_H, m (*J*, Hz)	^1H-^1H COSY	^1H-^{13}C HMBC
**4**	150.8			5, 1′
**5**	31.5, CH_2	2.39, dd (18.3, 3.8)<br>2.17, dd (18.3, 8.8)	5, 6<br>5, 6	4, 6, 7<br>4, 6, 7
**6**	75.3, CH	4.00, dddd (8.8, 7.8, 4.8, 3.8)	5, 7	5
**7**	33.7, CH_2	1.54, m<br>1.48, m	6, 8	5
**8**	24.3, CH_2	1.47, m<br>1.36, m	9, 7	
**9**	31.5, CH_2	1.27, m	8, 10	11
**10**	22.6, CH_2	1.30, m	9, 11	11
**11**	14.3, CH_3	0.87, t (6.8)	10	9, 10
**1′**	36.3, CH_2	2.23, t (7.3)<br>2.22, t (7.3)	2′	4, 2′
**2′**	25.3, CH_2	1.53, m	1′, 3′	1′
**3′**	31.2, CH_2	1.29, m	2′, 4′	5′
**4′**	22.4, CH_2	1.30, m	3′, 5′	5′
**5′**	14.3, CH_3	0.88, t (7.3)	4′	3′, 4′
**1′′**	165.6			2′′, 3′′
**2′′**	37.3, CH_2	2.69, m (6.6)<br>2.68, m (6.6)	3′′	1′′, 3′′, OH
**3′′**	57.4, CH_2	3.66, td (6.6, 5.4)	2′′, OH	1′′, 2′′, OH
**OH**		4.57, t (5.4)	3′′	2′′, 3′′

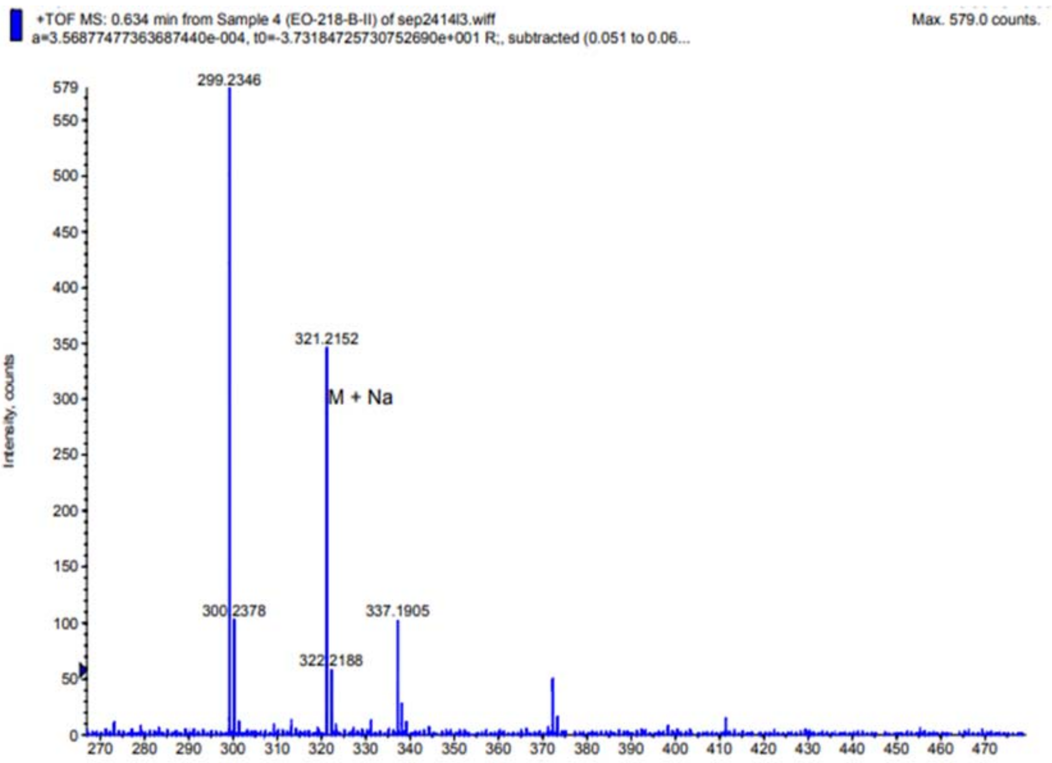

- **Figure S1**. HRESIMS spectrum of 1-[(6*R*)-5,6-Dihydro-4,6-dipentyl-2*H*-1,2,3-oxadiazin-2-yl]-3-hydroxypropan-1-one (**1**).

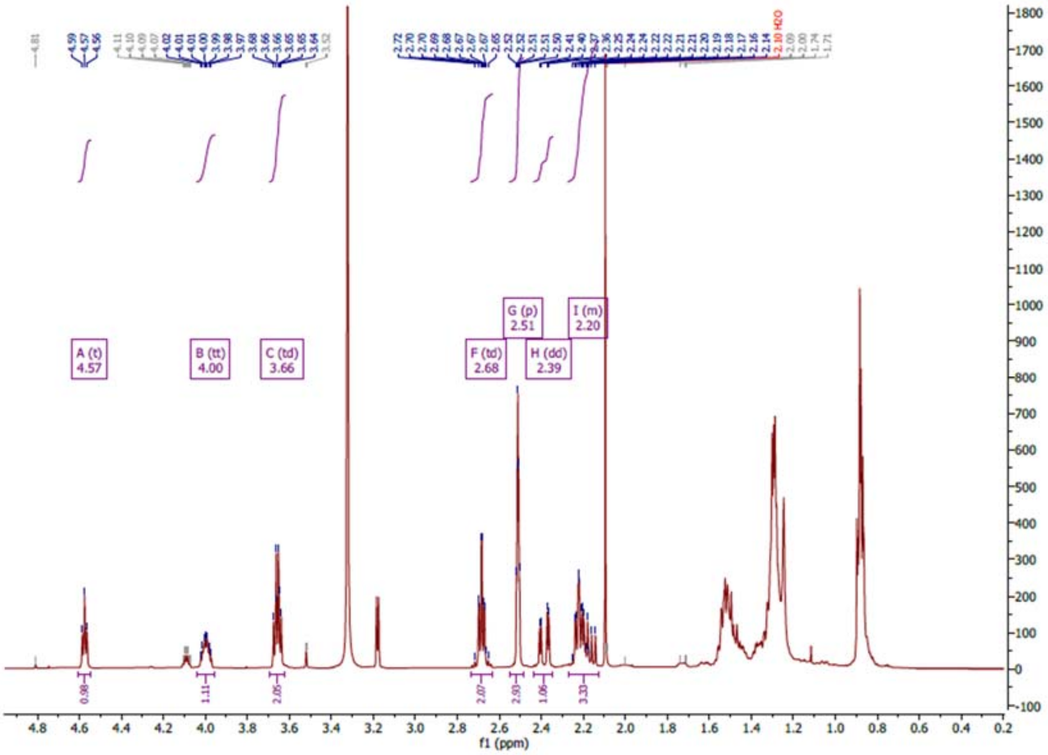

- **Figure S2**. ^{1}H NMR spectrum of 1-[(6*R*)-5,6-Dihydro-4,6-dipentyl-2*H*-1,2,3-oxadiazin-2-yl]-3-hydroxypropan-1-one (**1**) in DMSO 500 MHz.

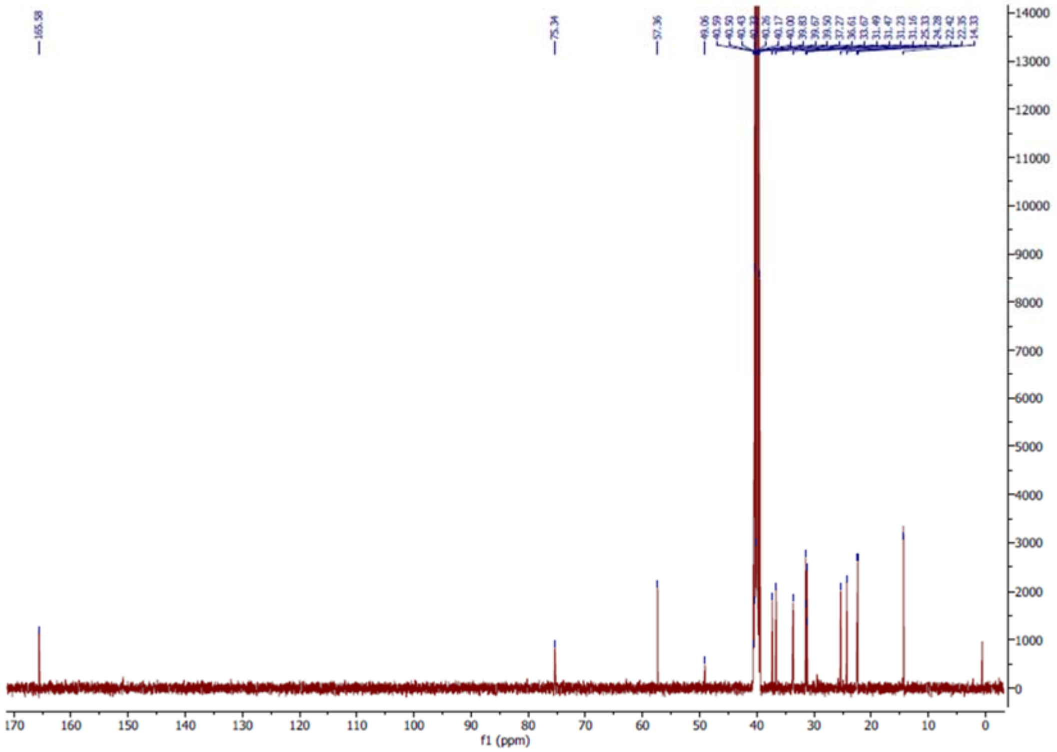

- **Figure S3**. ^{13}C NMR spectrum of 1-[(6*R*)-5,6-Dihydro-4,6-dipentyl-2*H*-1,2,3-oxadiazin-2-yl]-3-hydroxypropan-1-one (**1**) in DMSO 175 MHz.

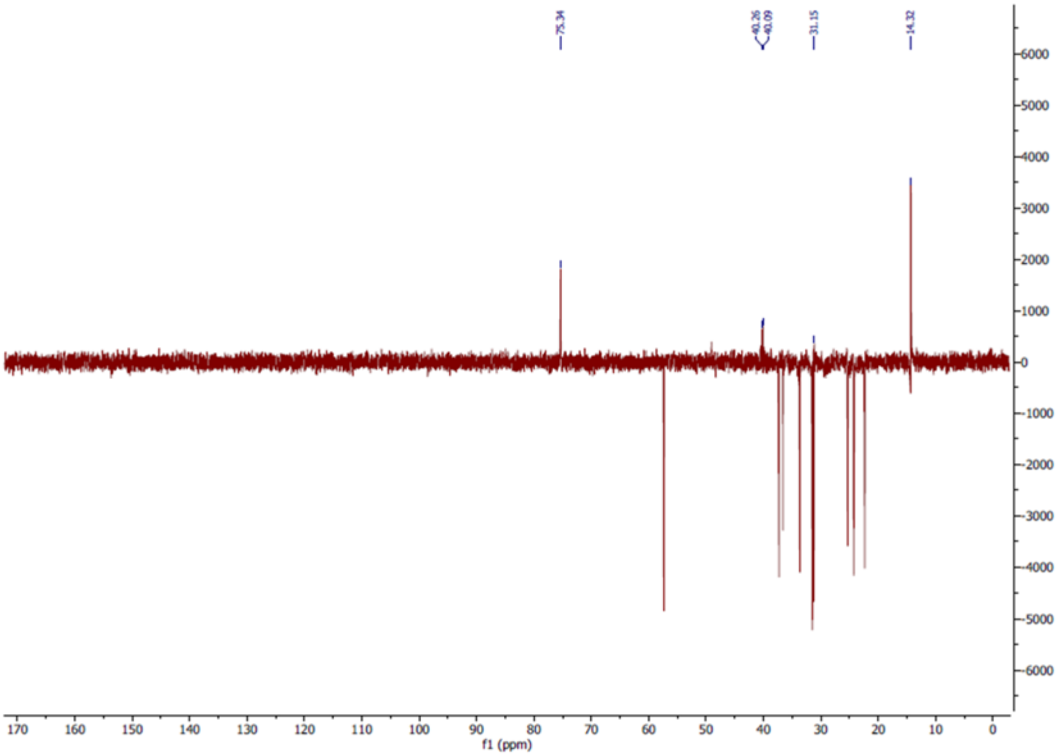

- **Figure S4**. DEPT-135 spectrum of 1-[(6*R*)-5,6-Dihydro-4,6-dipentyl-2*H*-1,2,3-oxadiazin-2-yl]-3-hydroxypropan-1-one (**1**) in DMSO 175 MHz.

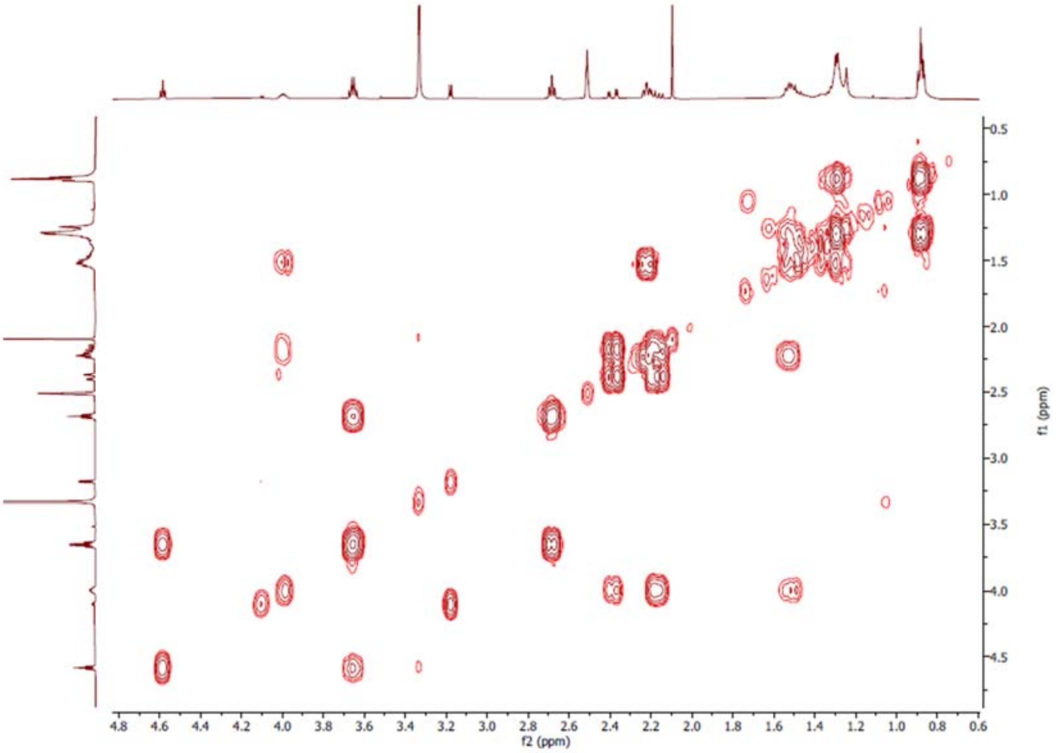

- **Figure S5**. ^{1}H-^{1}H COSY spectrum of 1-[(6*R*)-5,6-Dihydro-4,6-dipentyl-2*H*-1,2,3-oxadiazin-2-yl]-3-hydroxypropan-1-one (**1**) in DMSO.

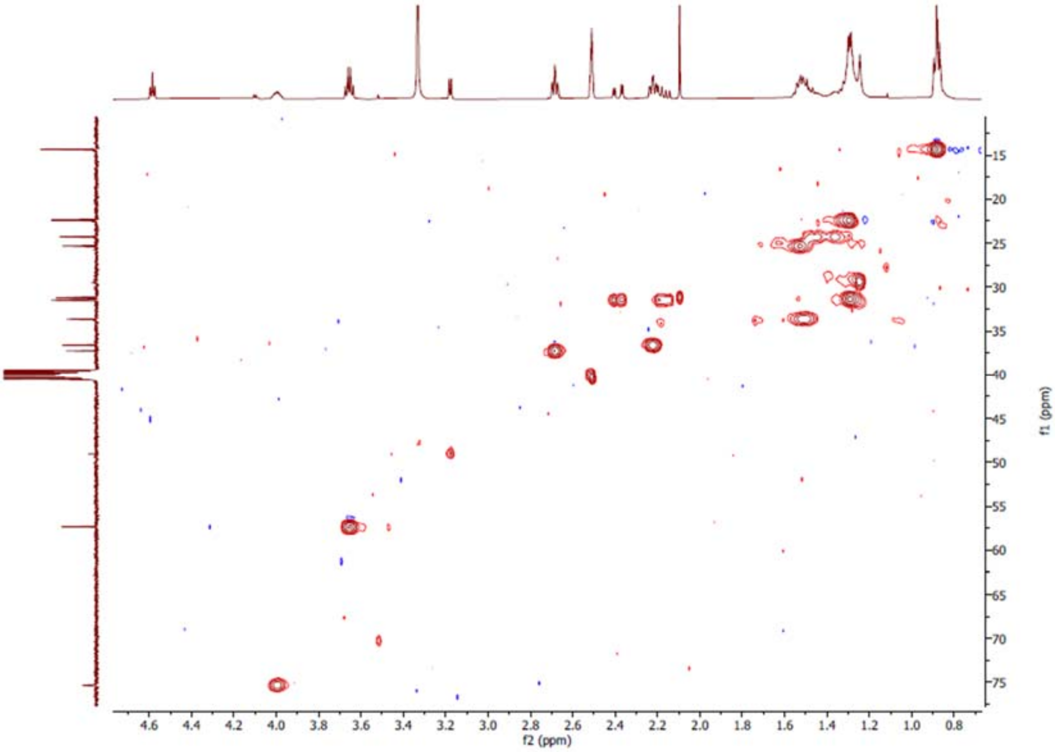

- **Figure S6**. ^{1}H-^{13}C HSQC spectrum of 1-[(6*R*)-5,6-Dihydro-4,6-dipentyl-2*H*-1,2,3-oxadiazin-2-yl]-3-hydroxypropan-1-one (**1**) in DMSO.

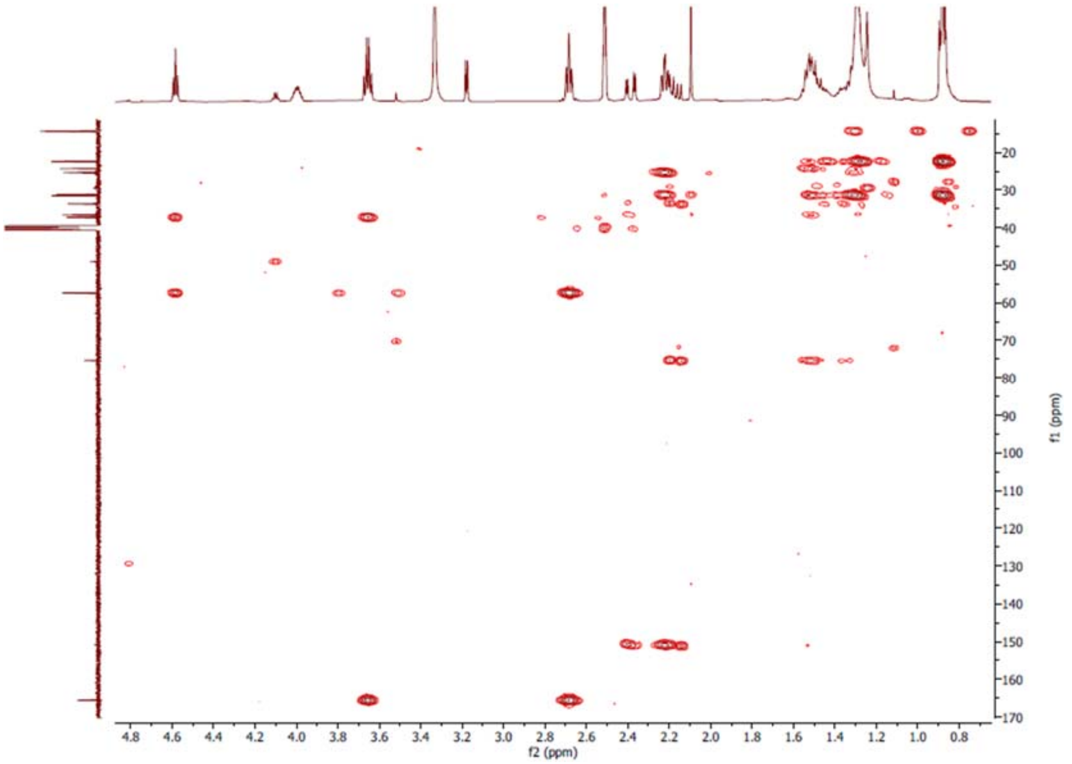

- **Figure S7**. ^{1}H-^{13}C HMBC spectrum of 1-[(6*R*)-5,6-Dihydro-4,6-dipentyl-2*H*-1,2,3-oxadiazin-2-yl]-3-hydroxypropan-1-one (**1**) in DMSO.

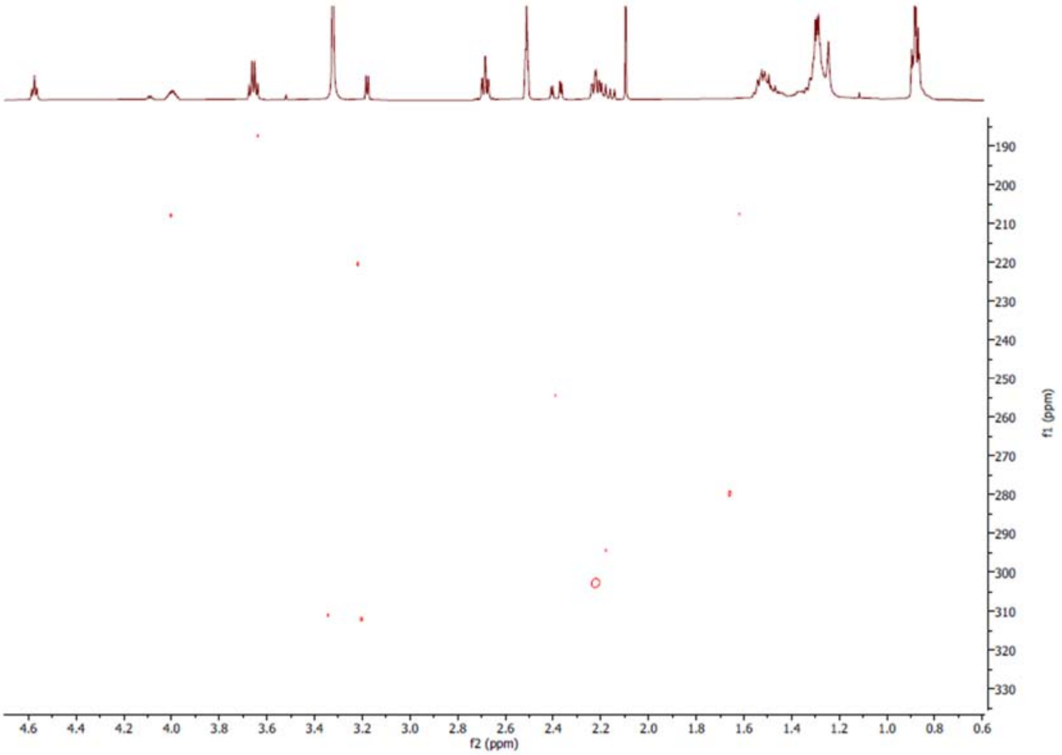

- **Figure S8**. ^{1}H-^{15}N HMBC spectrum of 1-[(6*R*)-5,6-Dihydro-4,6-dipentyl-2*H*-1,2,3-oxadiazin-2-yl]-3-hydroxypropan-1-one (**1**) in DMSO.

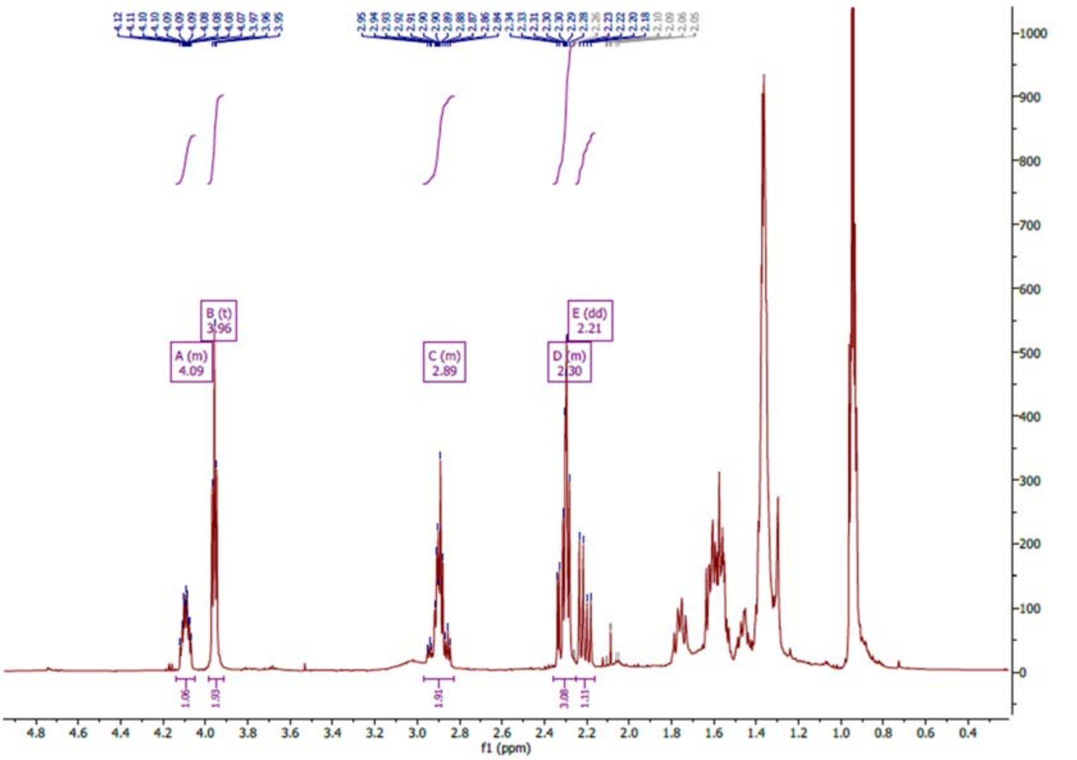

- **Figure S9**. ^{1}H NMR spectrum of 1-[(6*R*)-5,6-Dihydro-4,6-dipentyl-2*H*-1,2,3-oxadiazin-2-yl]-3-hydroxypropan-1-one (**1**) in $CDCl_3$ 500 MHz.

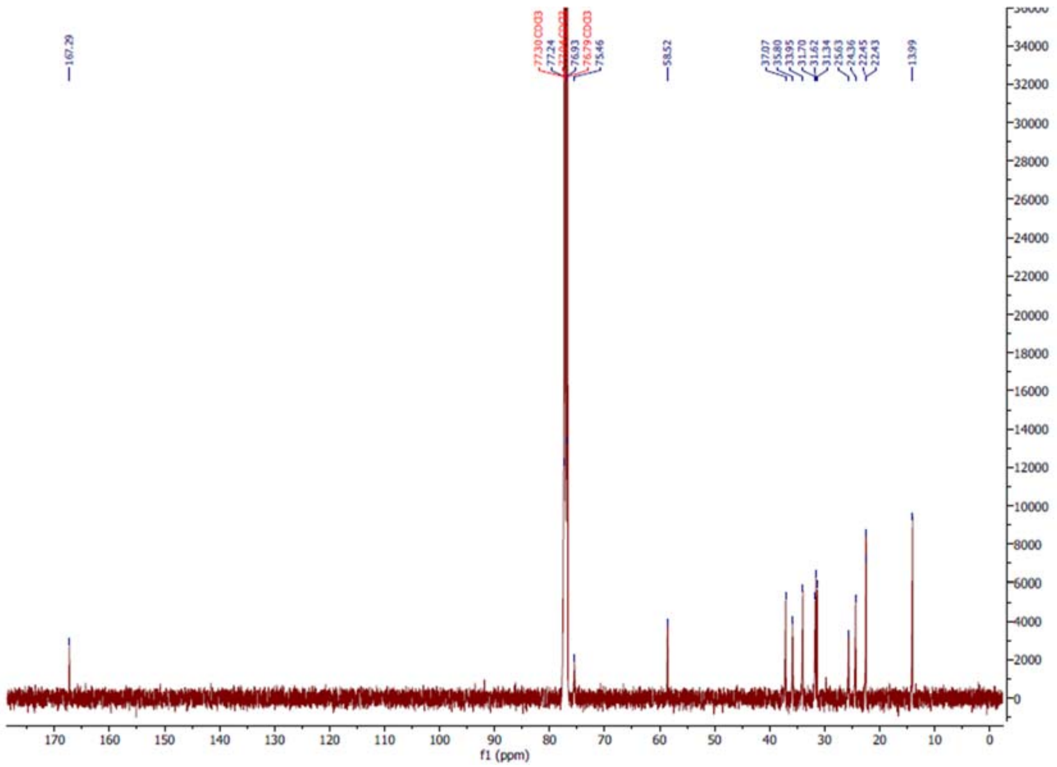

- **Figure S10**. ^{13}C NMR spectrum of 1-[(6*R*)-5,6-Dihydro-4,6-dipentyl-2*H*-1,2,3-oxadiazin-2-yl]-3-hydroxypropan-1-one (**1**) in $CDCl_3$ 175 MHz.

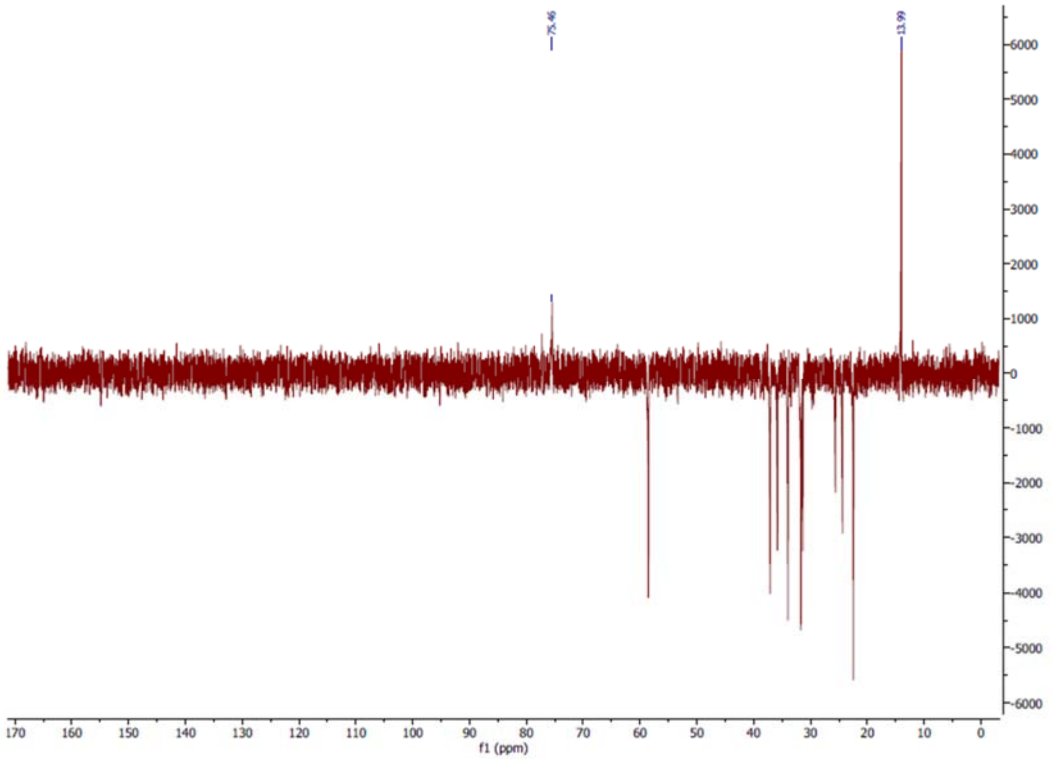

- **Figure S11**. DEPT-135 spectrum of 1-[(6*R*)-5,6-Dihydro-4,6-dipentyl-2*H*-1,2,3-oxadiazin-2-yl]-3-hydroxypropan-1-one (**1**) in $CDCl_3$ 175 MHz.

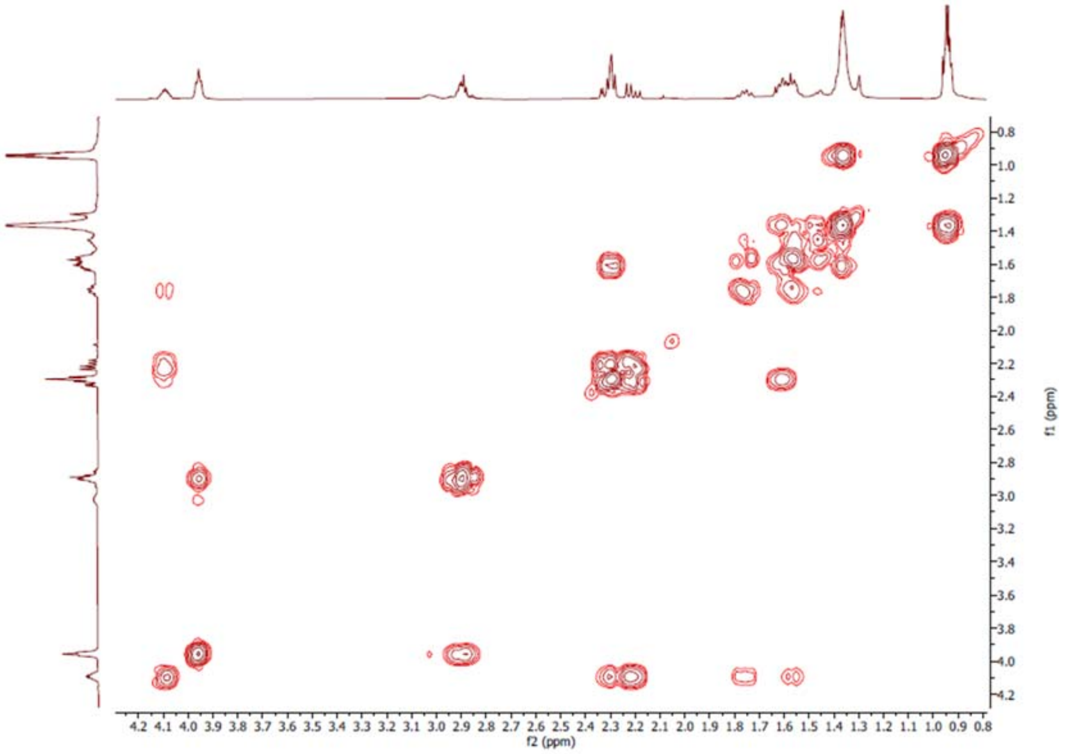

- **Figure S12**. ^1H-^1H COSY spectrum of 1-[(6*R*)-5,6-Dihydro-4,6-dipentyl-2*H*-1,2,3-oxadiazin-2-yl]-3-hydroxypropan-1-one (**1**) in $CDCl_3$.

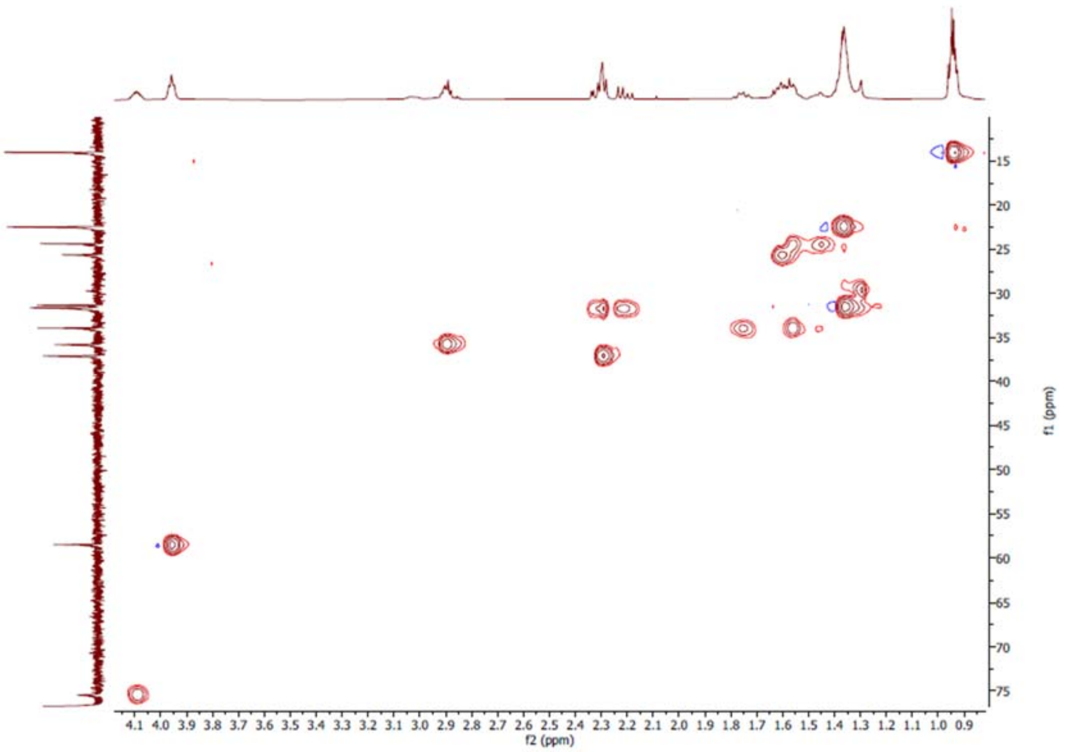

- **Figure S13**. ^{1}H-^{13}C HSQC spectrum of 1-[(6*R*)-5,6-Dihydro-4,6-dipentyl-2*H*-1,2,3-oxadiazin-2-yl]-3-hydroxypropan-1-one (**1**) in $CDCl_3$.

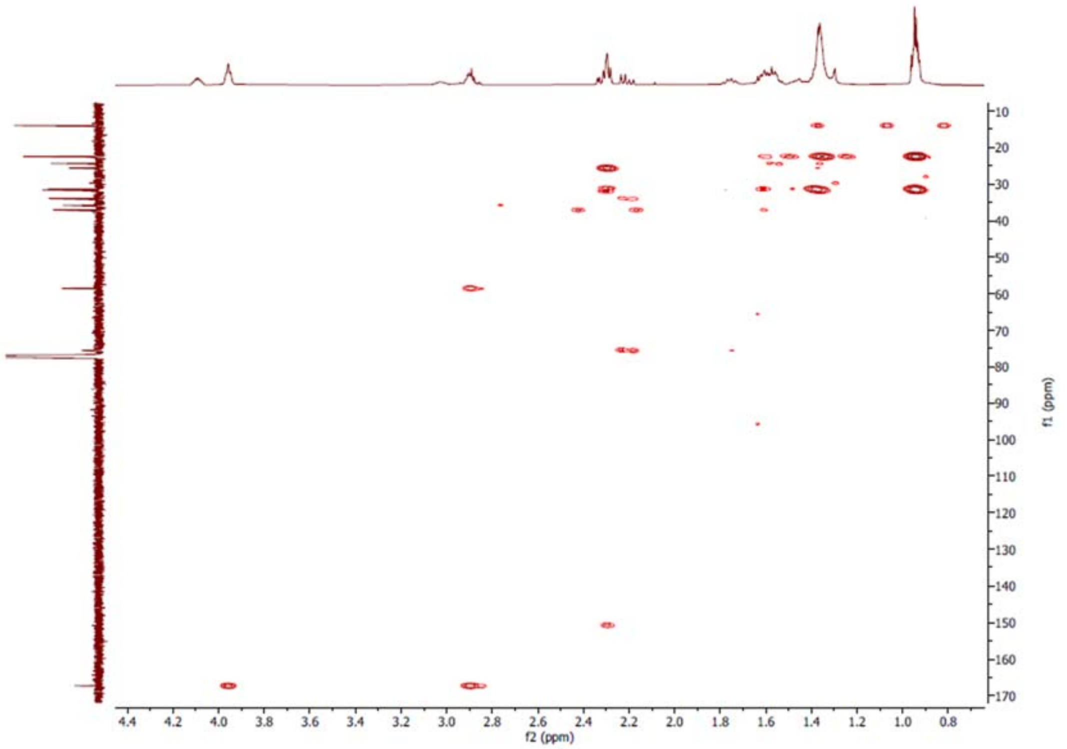

- **Figure S14**. ^{1}H-^{13}C HMBC spectrum of 1-[(6*R*)-5,6-Dihydro-4,6-dipentyl-2*H*-1,2,3-oxadiazin-2-yl]-3-hydroxypropan-1-one (**1**) in $CDCl_3$.

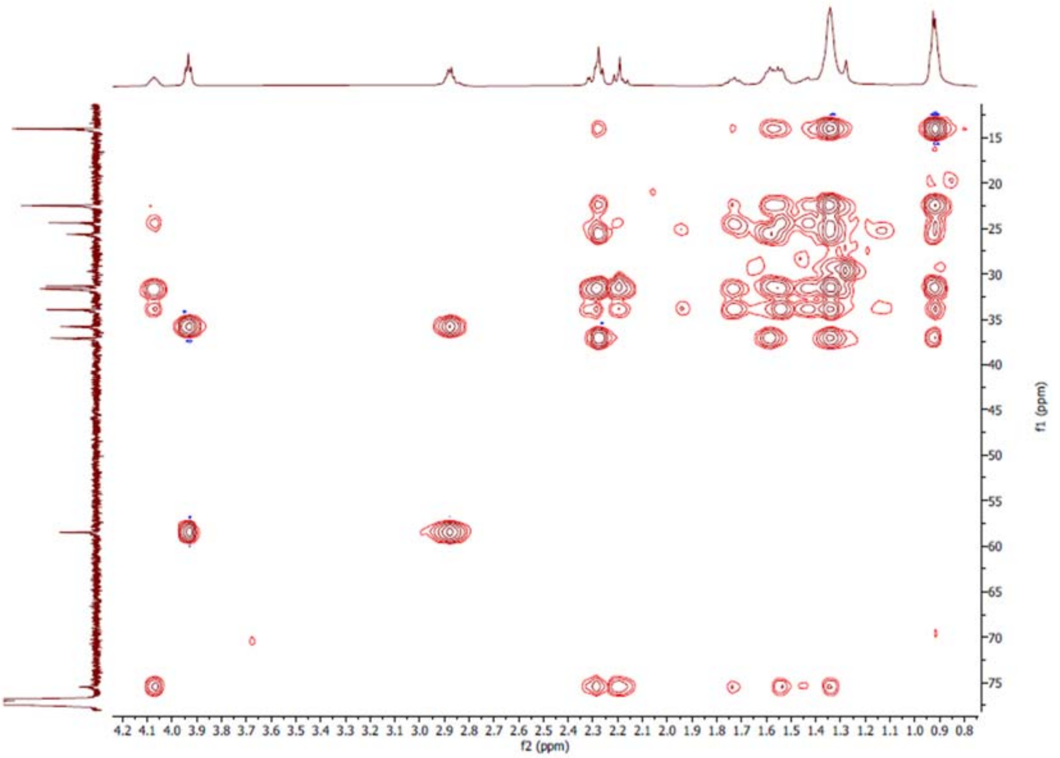

- **Figure S15**. ^{1}H-^{13}C HSQC-TOCSY spectrum of 1-[(6*R*)-5,6-Dihydro-4,6-dipentyl-2*H*-1,2,3-oxadiazin-2-yl]-3-hydroxypropan-1-one (**1**) in $CDCl_3$.

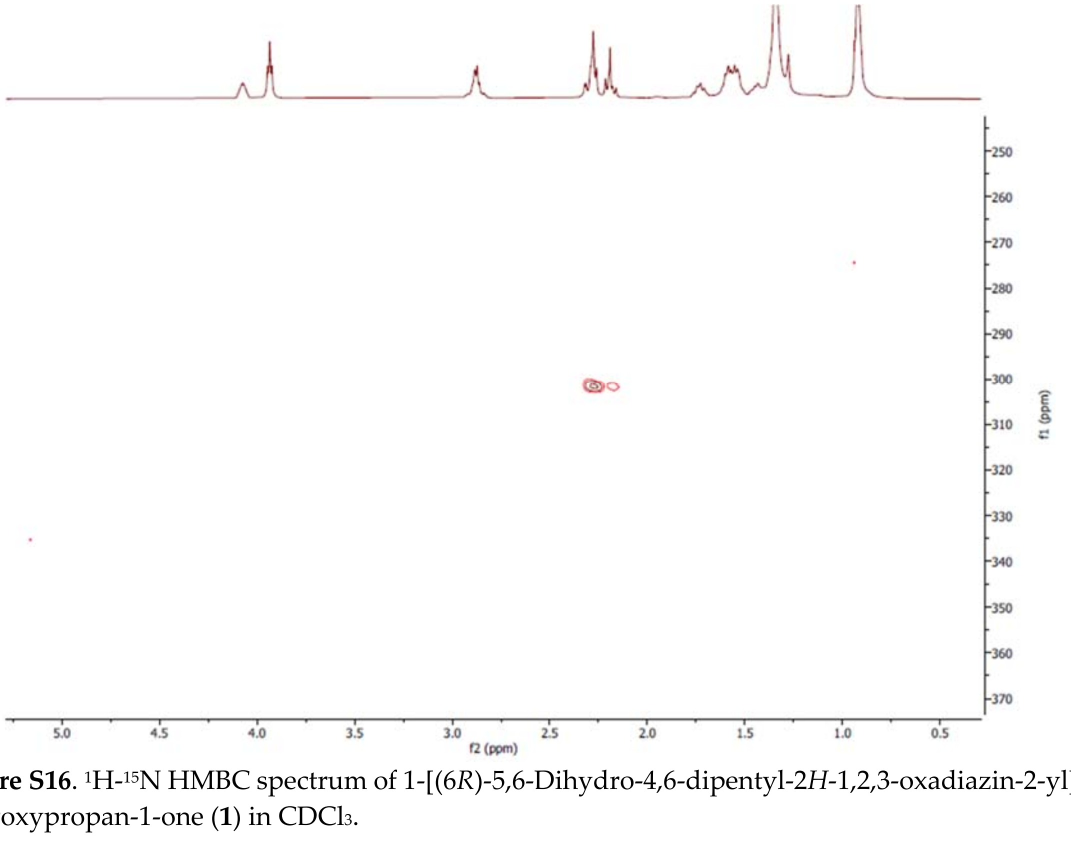

- **Figure S16**. ^{1}H-^{15}N HMBC spectrum of 1-[(6*R*)-5,6-Dihydro-4,6-dipentyl-2*H*-1,2,3-oxadiazin-2-yl]-3-hydroxypropan-1-one (**1**) in $CDCl_3$.

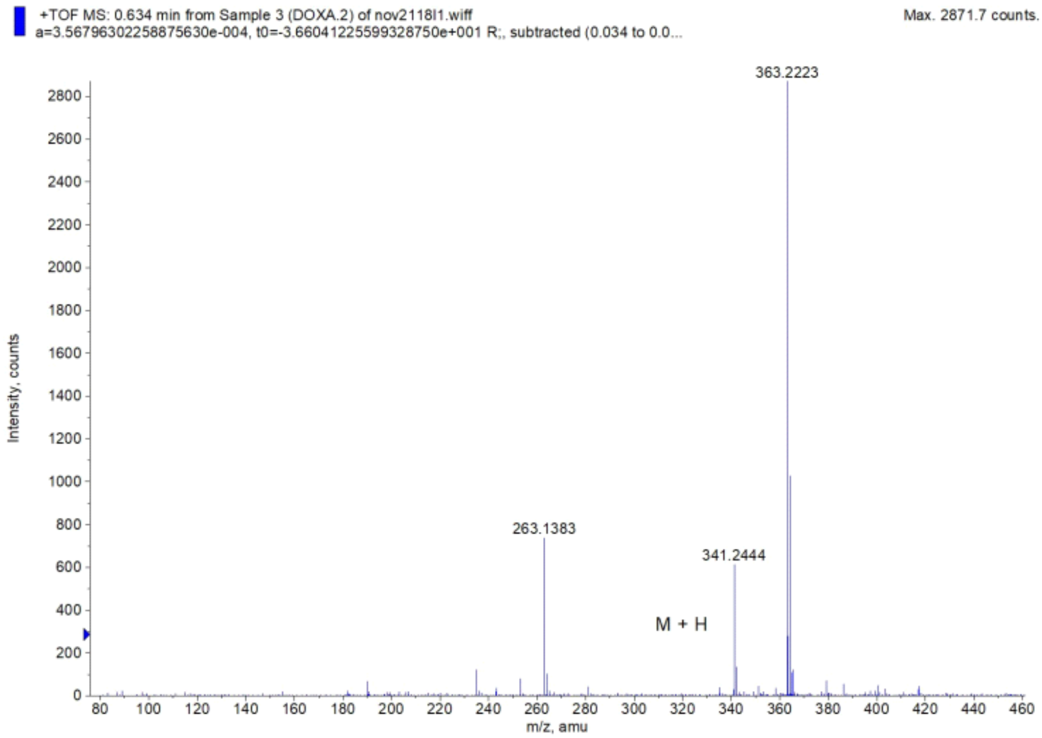

- **Figure S17**. HRESIMS spectrum of 3-[(6*R*)-5,6-Dihydro-4,6-dipentyl-2*H*-1,2,3-oxadiazin-2-yl]-3-oxopropyl acetate (**2**).

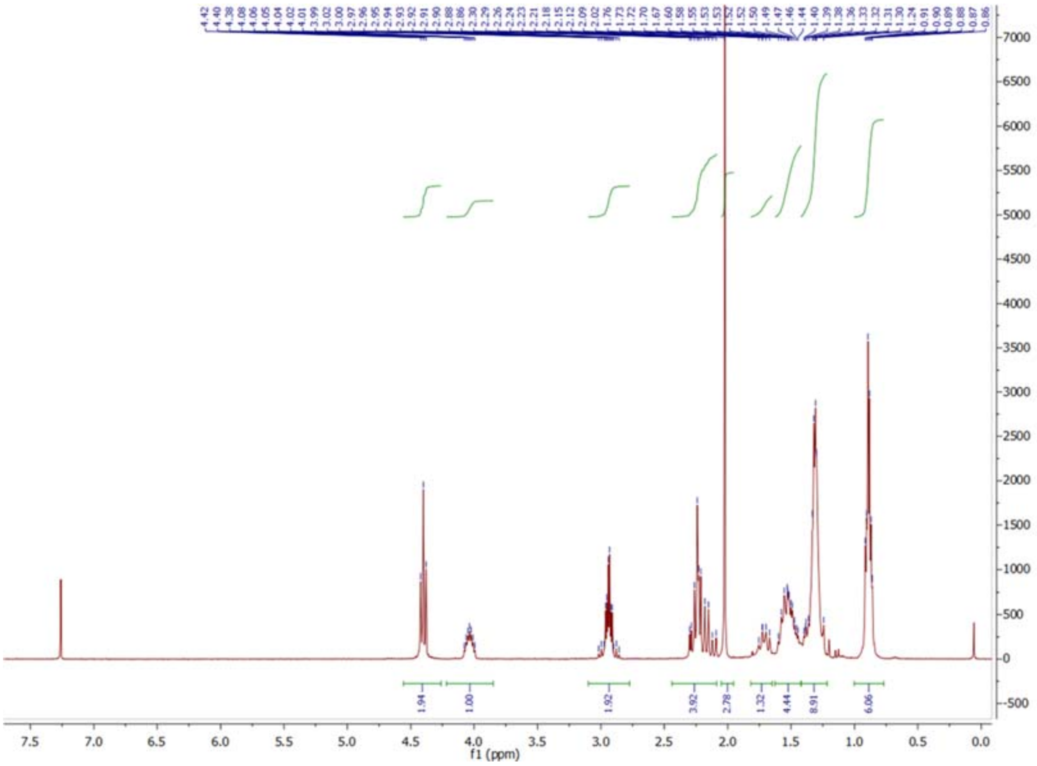

- **Figure S18**. ^{1}H NMR spectrum of 3-[(6*R*)-5,6-Dihydro-4,6-dipentyl-2*H*-1,2,3-oxadiazin-2-yl]-3-oxopropyl acetate (**2**) in $CDCl_3$ 300 MHz.

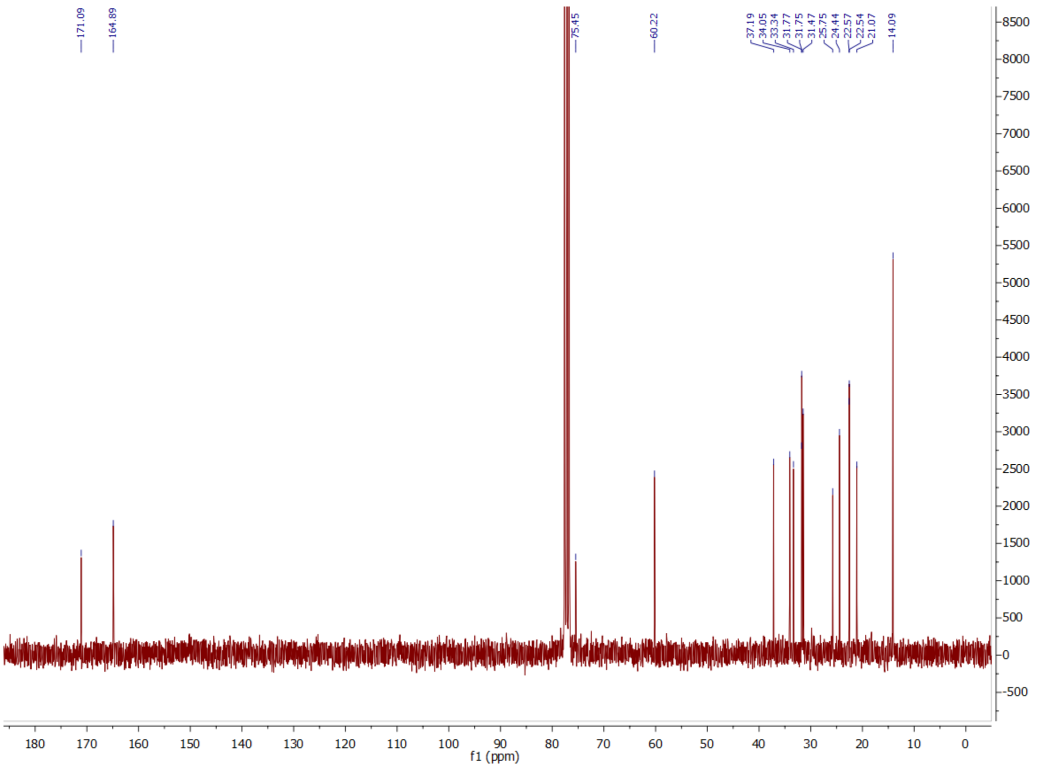

- **Figure S19**. ^{13}C NMR spectrum of 3-[(6*R*)-5,6-Dihydro-4,6-dipentyl-2*H*-1,2,3-oxadiazin-2-yl]-3-oxopropyl acetate (**2**) in $CDCl_3$ 75 MHz.

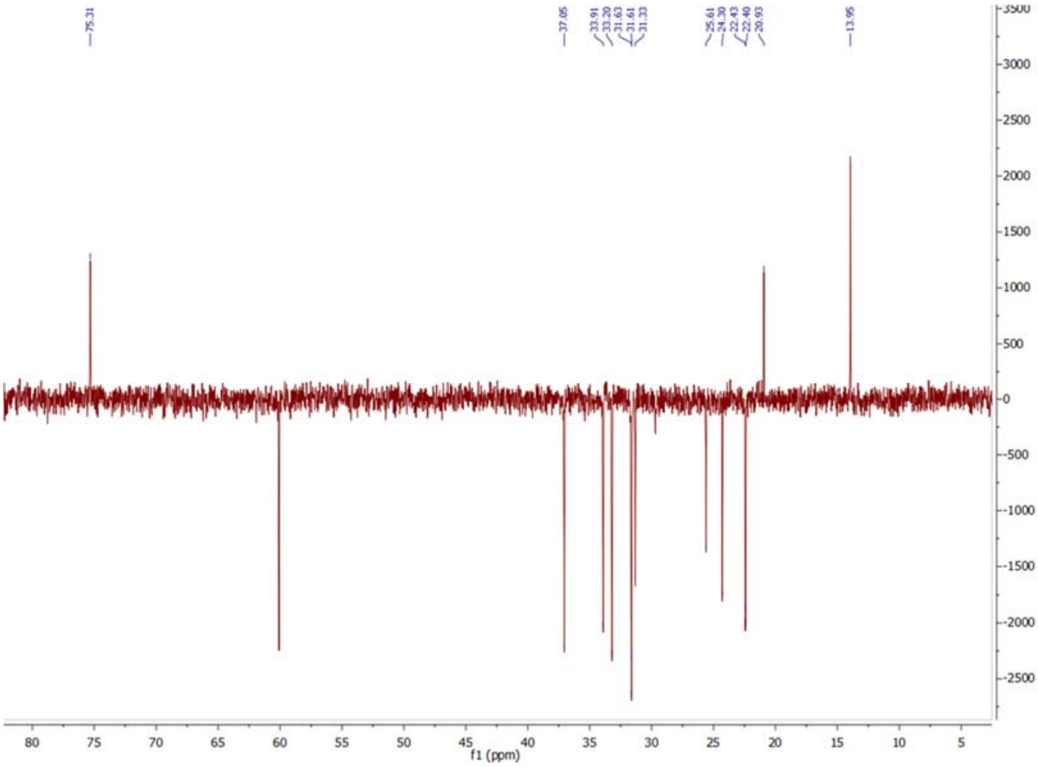

- **Figure S20**. DEPT-135 spectrum of 3-[(6*R*)-5,6-Dihydro-4,6-dipentyl-2*H*-1,2,3-oxadiazin-2-yl]-3-oxopropyl acetate (**2**) in $CDCl_3$ 75 MHz.

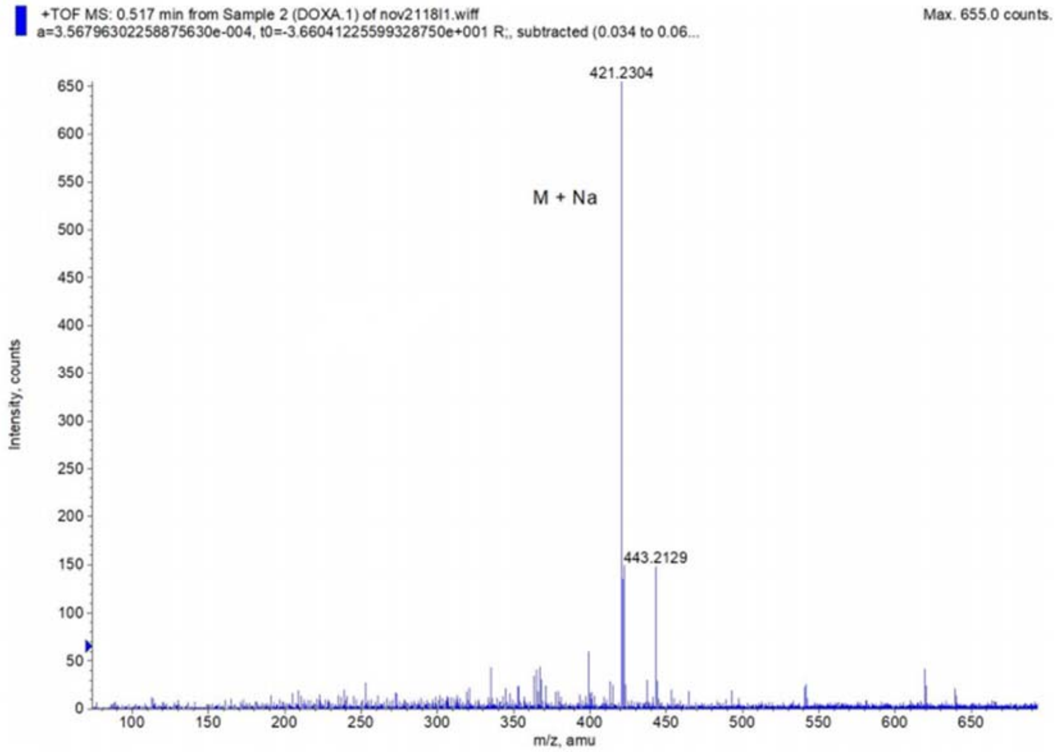

- **Figure S21**. HRESIMS spectrum of 4-{3-[(6*R*)-5,6-Dihydro-4,6-dipentyl-2*H*-1,2,3-oxadiazin-2-yl]-3-oxopropoxy}-4-oxobutanoic acid (**3**).

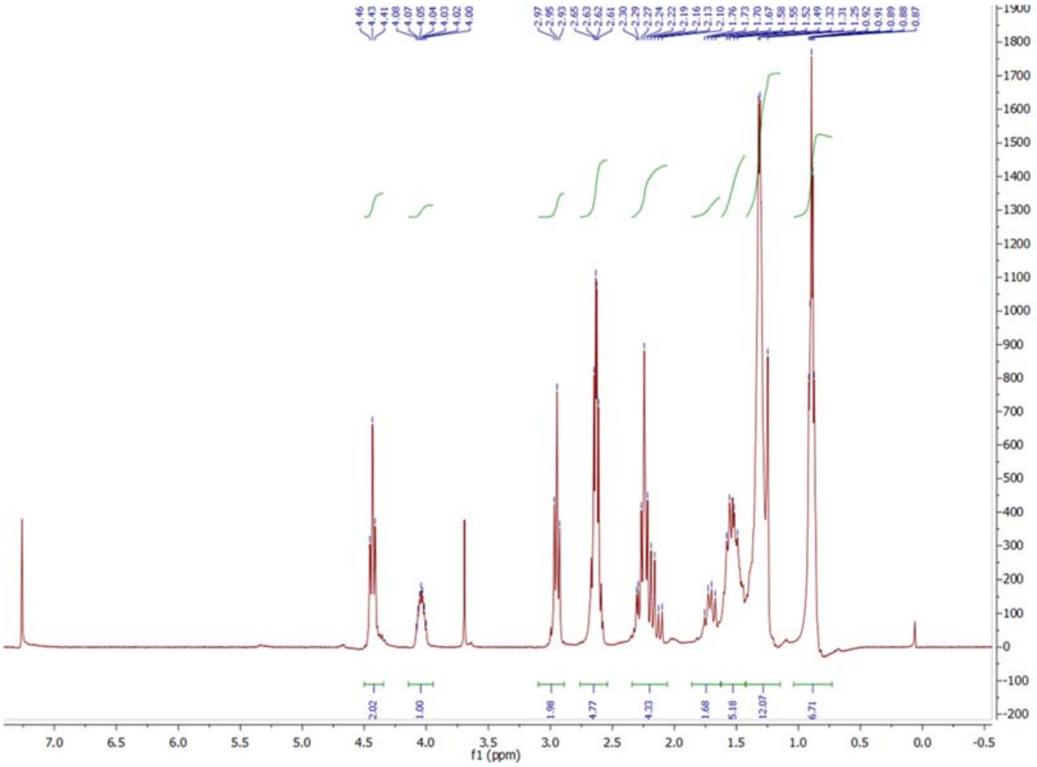

- **Figure S22**. ^{1}H NMR spectrum of 4-{3-[(6*R*)-5,6-Dihydro-4,6-dipentyl-2*H*-1,2,3-oxadiazin-2-yl]-3-oxopropoxy}-4-oxobutanoic acid (**3**) in $CDCl_3$ 300 MHz.

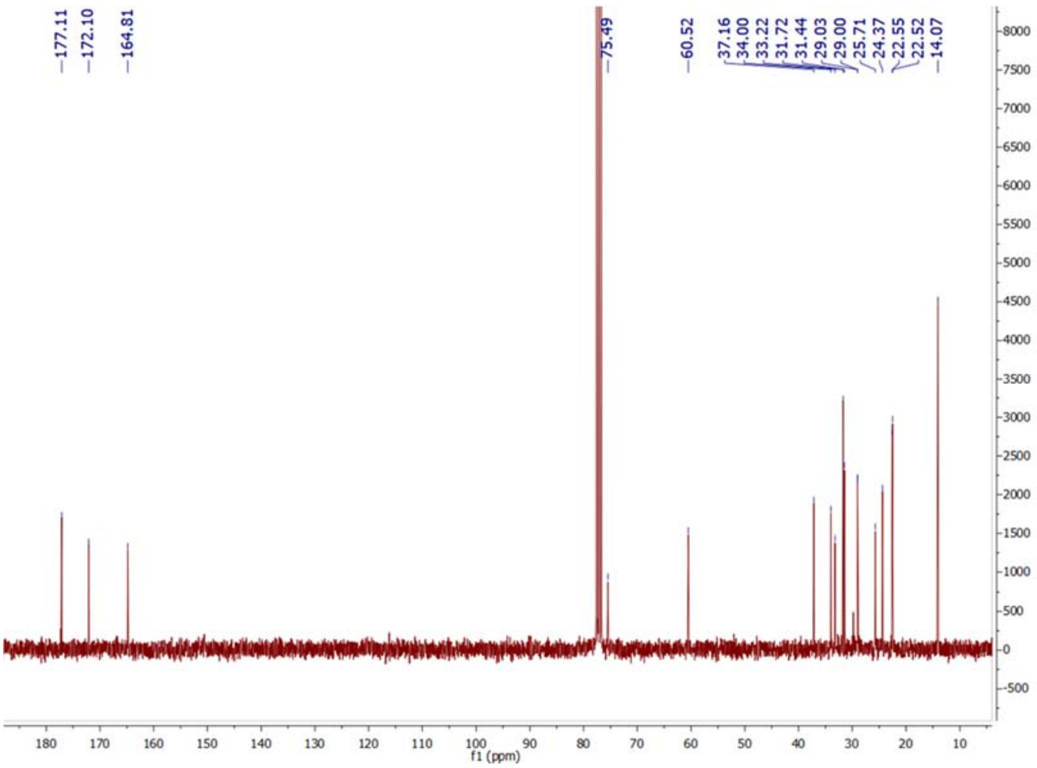

- **Figure S23**. ^{13}C NMR spectrum of 4-{3-[(6*R*)-5,6-Dihydro-4,6-dipentyl-2*H*-1,2,3-oxadiazin-2-yl]-3-oxopropoxy}-4-oxobutanoic acid (**3**) in $CDCl_3$ 75 MHz.

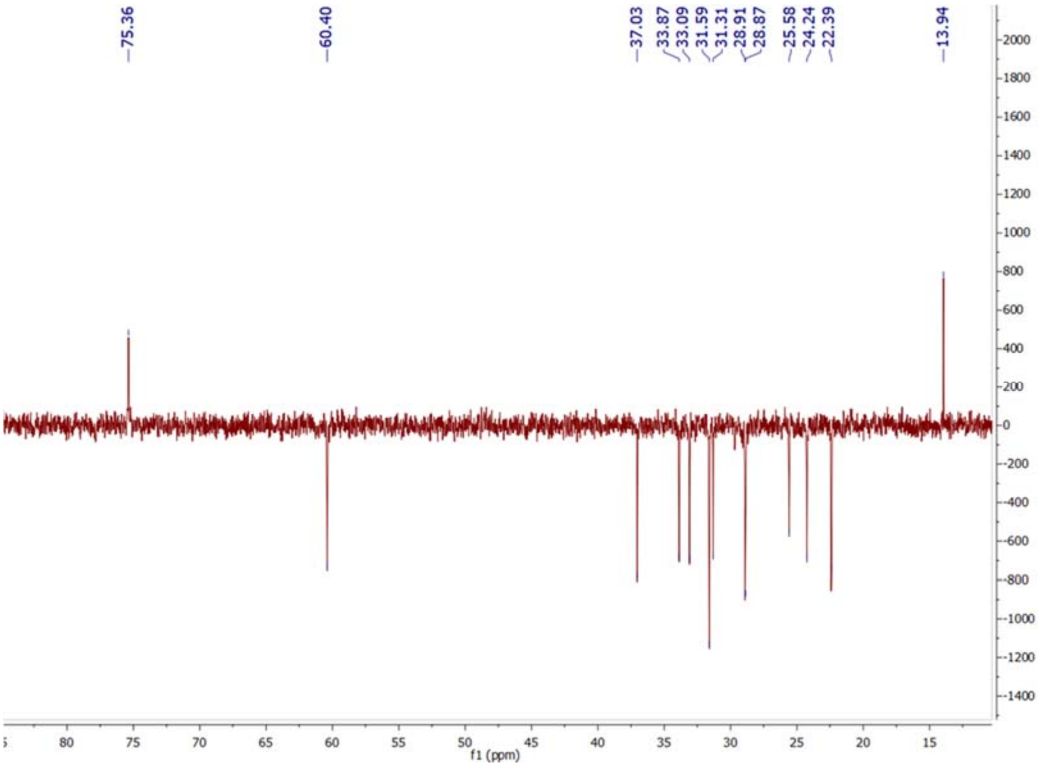

- **Figure S24**. DEPT-135 spectrum of 4-{3-[(6*R*)-5,6-Dihydro-4,6-dipentyl-2*H*-1,2,3-oxadiazin-2-yl]-3-oxopropoxy}-4-oxobutanoic acid (**3**) in $CDCl_3$ 75 MHz.

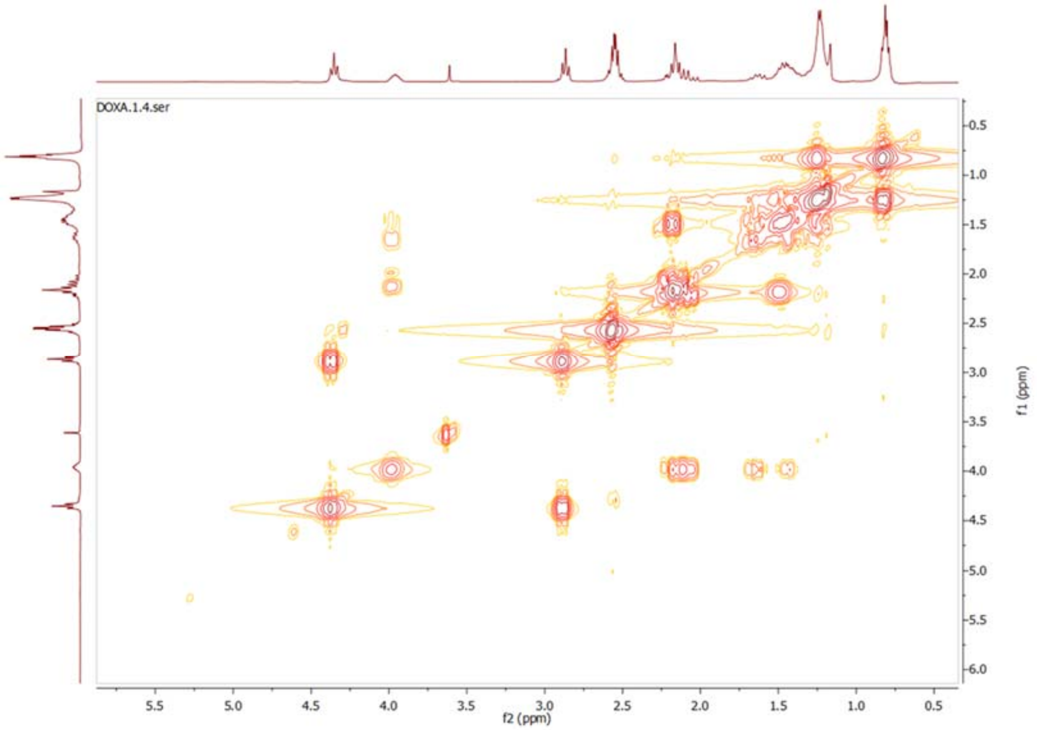

- **Figure S25**. ^{1}H-^{1}H COSY spectrum of 4-{3-[(6*R*)-5,6-Dihydro-4,6-dipentyl-2*H*-1,2,3-oxadiazin-2-yl]-3-oxopropoxy}-4-oxobutanoic acid (**3**) in $CDCl_3$.

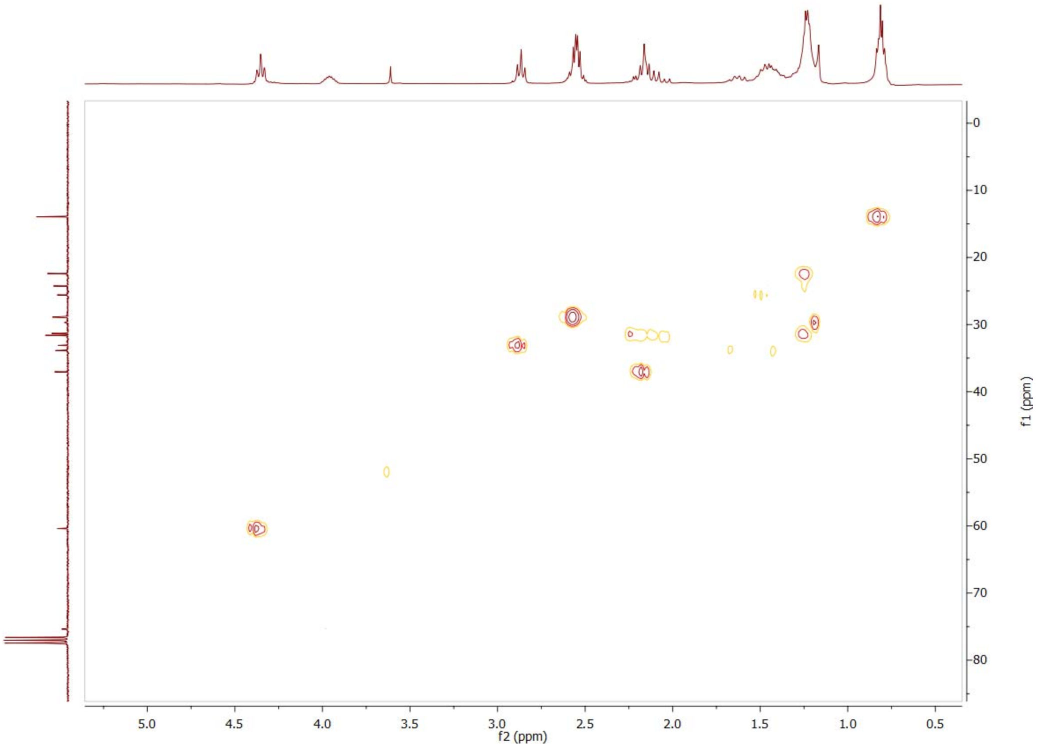

- **Figure S26**. ^{1}H-^{13}C HSQC spectrum of 4-{3-[(6*R*)-5,6-Dihydro-4,6-dipentyl-2*H*-1,2,3-oxadiazin-2-yl]-3-oxopropoxy}-4-oxobutanoic acid (**3**) in $CDCl_3$.

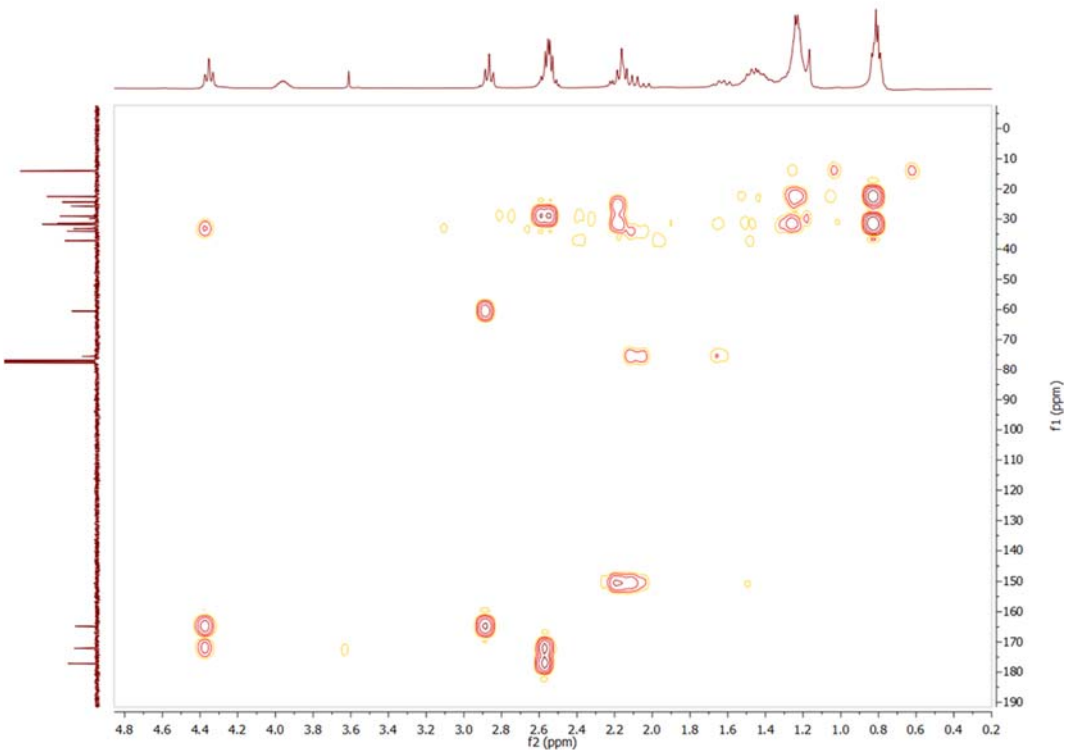

- **Figure S27**. ^{1}H-^{13}C HMBC spectrum of 4-{3-[(6*R*)-5,6-Dihydro-4,6-dipentyl-2*H*-1,2,3-oxadiazin-2-yl]-3-oxopropoxy}-4-oxobutanoic acid (**3**) in $CDCl_3$.
